# Integrating phylogeny, geographic niche partitioning and secondary metabolite synthesis in bloom-forming Planktothrix

**DOI:** 10.1038/ismej.2014.189

**Published:** 2014-10-17

**Authors:** Rainer Kurmayer, Judith F Blom, Li Deng, Jakob Pernthaler

**Affiliations:** 1 Research Institute for Limnology, University of Innsbruck Mondsee, Austria; 2 Limnological Station, Institute of Plant Biology, University of Zürich Kilchberg, Switzerland

## Abstract

Toxic freshwater cyanobacteria form harmful algal blooms that can cause acute toxicity to humans and livestock. Globally distributed, bloom-forming cyanobacteria *Planktothrix* either retain or lose the *mcy* gene cluster (encoding the synthesis of the secondary metabolite hepatotoxin microcystin or MC), resulting in a variable spatial/temporal distribution of (non)toxic genotypes. Despite their importance to human well-being, such genotype diversity is not being mapped at scales relevant to nature. We aimed to reveal the factors influencing the dispersal of those genotypes by analyzing 138 strains (from Europe, Russia, North America and East Africa) for their (i) *mcy* gene cluster composition, (ii) phylogeny and adaptation to their habitat and (iii) ribosomally and nonribosomally synthesized oligopeptide products. Although all the strains from different species contained at least remnants of the *mcy* gene cluster, various phylogenetic lineages evolved and adapted to rather specific ecological niches (for example, through pigmentation and gas vesicle protein size). No evidence for an increased abundance of specific peptides in the absence of MC was found. MC and peptide distribution rather depended on phylogeny, ecophysiological adaptation and geographic distance. Together, these findings provide evidence that MC and peptide production are primarily related to speciation processes, while within a phylogenetic lineage the probability that strains differ in peptide composition increases with geographic distance.

## Introduction

For decades, harmful algal blooms, formed by freshwater cyanobacteria, have been linked to acute toxicity to humans and livestock (Hudnell, 2008). The best-characterized cyanotoxin is the hepatotoxin microcystin (MC), produced mainly by the planktonic genera *Anabaena*, *Planktothrix* and *Microcystis*. The genus *Planktothrix* (Suda *et al.*, 2002) is globally distributed and abundant in lakes and reservoirs (Kurmayer *et al.*, 2011).

Elucidation of the genetic basis of the synthesis of MC and other more frequent cyanotoxins (Kurmayer and Christiansen, 2009; Dittmann *et al.*, 2013) made way for investigating the evolution of these compounds. Within each genus of cyanobacteria, toxic strains that are able to produce a certain toxin always coexist with nontoxic strains, which was hypothesized to be due to horizontal gene transfer (HGT; for example, Nakasugi *et al.*, 2007). However, the genes encoding biosynthetic pathway of MCs and the closely related nodularin evolved at the same rate as housekeeping genes (for example, 16S rRNA and *rpo*C1). Therefore, a common MC-producing ancestor was proposed (Rantala *et al.*, 2004). We previously characterized nontoxic *Planktothrix* strains that lost >90% of the *mcy* gene cluster encoding MC synthesis (herein termed strains that lost the *mcy* gene cluster, Christiansen *et al.*, 2008a). Correspondingly, in another genus, nontoxic *Microcystis* strains lost a major part of the *mcy* gene cluster (Tooming-Klunderud *et al.*, 2008), and the same two genes (*dna*N and *uma*1) flank the remnant or complete *mcy* gene cluster in nontoxic or toxic strains. These findings support the occurrence of gene loss rather than HGT of *mcy* genes in *Planktothrix* and *Microcystis.*

Losing the *mcy* gene cluster in *Planktothrix* could have occurred millions of years ago (Christiansen *et al.*, 2008a). Two lineages were identified based on Multi Locus Sequence Typing (MLST) using four housekeeping genes: lineage 1 comprised strains that lost the *mcy* gene cluster, and lineage 2 comprised strains still containing the full *mcy* gene cluster. Surprisingly, lineage 1 also contained a number of strains that retained the *mcy* gene cluster, suggesting that the loss of toxicity *per se* did not lead to phylogenetic diversification. Due to this rather long history of *mcy* gene loss, it is hypothesized that *Planktothrix* meanwhile could have adapted to a variety of ecological factors not causally related to MC synthesis. For example, the strains of toxic lineage 2 contained many red-pigmented strains assigned to *P. rubescens*, which generally have resistance to high hydrostatic pressure under deep-mixing conditions due to adaptation in gas vesicle protein size when compared with green-pigmented *P. agardhii* (Beard *et al.*, 2000).

The acute toxicity of MC to various aquatic biota contributes to the deterrence of grazers (Kurmayer and Jüttner, 1999) and parasites (Rohrlack *et al.*, 2013), although various physiological functions of MC have been suggested (Neilan *et al.*, 2013). The loss of MC production for individual strains was suggested to be functionally compensated by other bioactive oligopeptides, for example, peptides inhibiting digestive enzymes, such as serine proteases (trypsin) of herbivores (Rohrlack *et al.*, 2005). Oscillapeptin J, a member of the family of the cyanopeptolins, has been described as an effective inhibitor of chymotrypsin and trypsin and showed comparable toxicity against herbivores compared with the major MC structural variant purified from the same strain of *P. rubescens* (Blom *et al.*, 2006). Similarly, planktocyclin isolated from a *P. rubescens* bloom was also described as a strong (chymo)trypsin inhibitor (Baumann *et al.*, 2007). Although cyanopeptolins are synthesized nonribosomally through large multifunctional peptide synthetases or nonribosomal peptide synthetases (Rounge *et al.*, 2007), the planktocyclins are likely produced ribosomally through posttranslational modification (Ziemert *et al.*, 2008). Thus two different biosynthetic routes form bioactive peptides inhibiting digestive enzymes with comparable efficiency within individual strains.

Here we developed a framework for an approach of cyanobacterial ecology based on phylogeny, niche partitioning and bioactive peptides and used it to address the question of how cyanobacterial individuals adapt in diverse environments. *Planktothrix* strains isolated from Europe, Russia, North America and East Africa (Table 1; Supplementary Table S1) were analyzed for (i) the presence of the *mcy* gene cluster remnants in nontoxic strains, (ii) the genetic variation within seven housekeeping gene loci and (iii) the occurrence of MC and other potentially bioactive oligopeptides. Such wide geographic sampling is necessary to reveal the global dispersal of clonal lineages that either lost or retained the *mcy* gene cluster. Because clonal dependence can be observed, if ecological differentiation prevents genetic exchange and, therefore, favors genetic differentiation (Cohan, 2002), *vice versa* information on the dispersal of individual lineages provides information on the ecological factors contributing to diversification. It is important to know which additional oligopeptides might functionally replace the lost MC. Finally, comparing the frequency of peptide occurrence between lineages will show which peptides could be related to the observed speciation processes.

## Materials and methods

Detailed methods for sample processing and analysis are provided in Supplementary Information.

### Organisms and genetic analysis of strains

All clonal *Planktothrix* strains were isolated by cutting out single filaments migrating on agar as described (Kurmayer *et al.*, 2004), and cultivated at 15 °C under low light conditions (5–10 μmol m^−2^ s^−1^) in BG11 medium (Rippka, 1988). In total, 138 strains were analyzed, including 62 isolated previously (Christiansen *et al.*, 2008a); Table 1; Supplementary Table S1). Strains were analyzed for (i) the presence of the full *mcy* gene cluster and the *mcy*T gene, which occurred in toxic strains and as a remainder in nontoxic strains (Christiansen *et al.*, 2008a), (ii) the genetic variation within seven housekeeping gene loci and intergenic spacer regions (IGS): 16S rDNA, 16S rDNA-internal transcribed spacer region (ITS), phycocyanin (PC)-IGS, photosystem I (PSA)-IGS (in between photosystem I related *psa*A and *psa*B), RNaseP, *rbc*LX-IGS (in between the large subunit of the ribulose bisphosphate carboxylase/oxygenase and *rbc*X), and *rpoC*. In addition, the ability to express gas vesicle protein variants of 28, 20 and 16 kDa was revealed by the presence of the genes *gvp*C^28^, *gvp*C^20^ and *gvp*C^16^ as described (Beard *et al.*, 2000; D'Alelio *et al.*, 2011).

### Pigment and peptide analysis of strains

For each strain, phycocyanin (PC)/phycoerythrin (PE) ratios were determined in duplicate within a time interval of 6 months (Tandeau de Marsac, 1977). MC production was measured by two independent methods—the protein phosphatase 1A inhibition assay and high-performance liquid chromatography-diode array detection (Kurmayer *et al.*, 2004); and general oligopeptide analysis was conducted by separating the peptides on another high-performance liquid chromatography system directly coupled to Electrospray Ionization Mass Spectrometry (Baumann *et al.*, 2007).

### Phylogenetic analysis

Sequences of partial *mcy*T gene (398 bp, *n*=124) were aligned (Clustal W 1.8). For Multi Locus Sequence Analysis (MLSA), sequences of 16S rDNA, 16S rDNA-ITS, PC-IGS, PSA-IGS, RNaseP, *rbc*LX-IGS and *rpo*C (2697 bp, *n*=138) were concatenated and aligned. Nucleotide substitution parameters were estimated by Maximum likelihood analysis (BASEML of the PAML package; Yang, 1997). Ambiguous sites were removed and phylogenetic analysis was constructed using maximum likelihood (ML), neighbor-joining (NJ) and maximum parsimony in PHYLIP (Felsenstein, 1993), with a bootstrap analysis of 1000 replicates.

For MLST, all 138 strains were defined by the alleles (unique genotypes) present at the seven gene loci (the allelic profile), and each unique allelic profile (= sequence genotype) was assigned a sequence type (ST). Isolates with the same ST at all loci were members of a single clone (Feil *et al.*, 2004). The program eBurst (V3) was used on the MLST website (http://www.mlst.net/) to divide the strains into clonal complexes.

The Bayesian-based ClonalFrame tool (Didelot and Falush, 2007) was used to measure the frequency of recombination events happening relative to mutations (*ρ*/*θ*) within and between different clusters. ClonalFrame predicted clonal genealogy after 5000 burn-in iterations and 5000 iterations based on comparing genealogies of triplicate runs. Non-polarized McDonald–Kreitman selection test (Egea *et al.*, 2008) was calculated between remnant and functional *mcy*T genes. The number of synonymous and nonsynonymous substitutions was detected and used to calculate the Neutrality Index (NI) and evaluated using Chi-Square.

### Statistical analysis of peptide composition

Canonical Correspondence Analysis (CCA) was performed (CANOCO 5.0 for Windows, Ter Braak and Šmilauer, 2012) to determine the dependence of peptide occurrence on phylogenetic and environmental parameters (Supplementary Table S1). For this purpose, two matrices were constructed, (i) one containing the log (*x*+1) transformed variables (*n*=10) describing the phylogeny, ecophysiology (PC/PE ratio, *gvp*C^16^, *gvp*C^20^, *gvp*C^28^ gene presence) and environment (maximum depth, mean depth, area of the water body, catchment area and geographic distance) for each strain (*n*=127) or ST (*n*=60), and (ii) one containing the presence/absence data on peptide occurrence (*n*=95) for each strain (*n*=127) or ST (*n*=60).

## Results

### Identification of *mcy* gene cluster remnants

**Figure 1:**
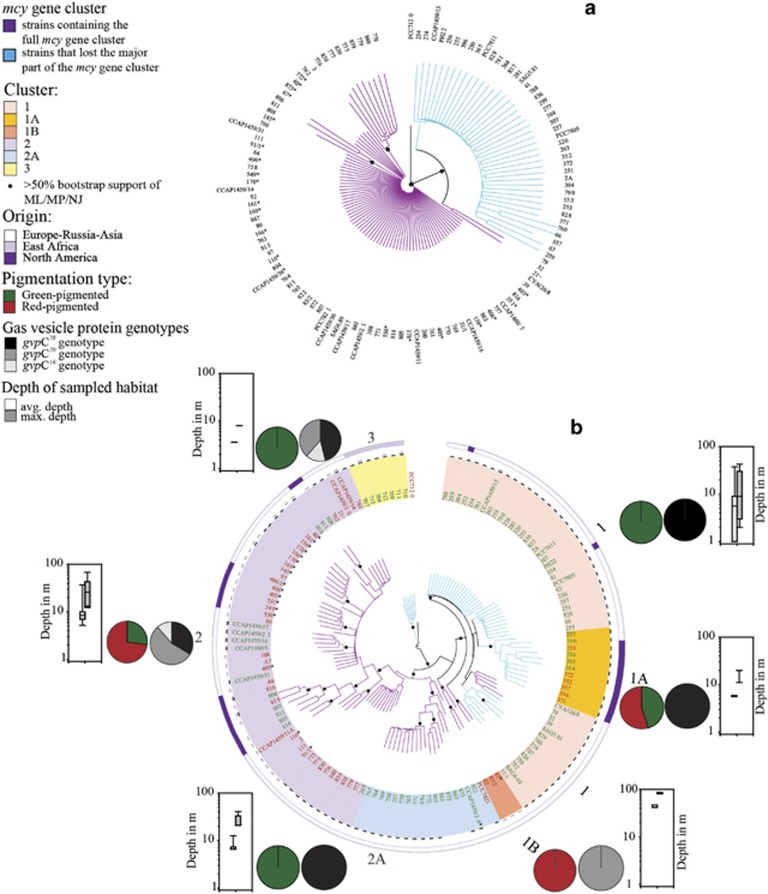
Maximum likelihood tree constructed from sequences of *Planktothrix* strains containing (**a**) *mcy*T both as part and remnant of the *mcy* gene cluster and (**b**) seven housekeeping genes and intergenic spacer regions. Nodes of >50% bootstrap values from maximum likelihood/maximum parsimony/neighbor-joining are indicated by filled black circles; the trees are rooted using *Nostoc* sp. strain PCC7120 as an outgroup; strains that either lost (blue) or retained (purple) the *mcy* gene cluster are indicated by colored branches; clusters of leaves are colored and marked by numbers: 1 (light orange), 1A (orange), 1B (darker orange), 2A (blue), 2 (purple), and 3 (yellow); green vs red pigmentation types are indicated by green or red color of the strain ID; gas vesicle protein genotypes are indicated by white (*gvp*C^16^), gray (*gvp*C^20^) and dark (*gvp*C^28^) bars; pie charts illustrate relative frequency of green vs red pigmentation types and gas vesicle protein genotypes assigned to phylogenetic lineages; box plots show mean (white bars) and maximum depth (grey bars) of sampled habitats for strains assigned to phylogenetic lineages; strains' origins are specified in the outer ring; see Supplementary Table S1 for strain details; *Strains that have been found containing the whole *mcy* gene cluster but found inactive.

All strains that did not contain a complete *mcy* gene cluster showed remnants of it (*n*=56; Table 2). The majority (*n*=42, 75%) contained full or partial *mcy*T (398 bp), which encodes a type II thioesterase shown to be involved in MC synthesis (Christiansen *et al.*, 2008a). The other 14 nontoxic strains contained the 5′ flanking region identical to the *mcy* gene cluster in *P. agardhii* toxic strain NIVA-CYA126/8 (AJ441056, Christiansen *et al.*, 2003, max. 1.1% dissimilarity, 646 bp) and 13 strains contained a smaller remnant of *mcy*T (169 bp). Strain No. 713 had a 202-bp insertion that was identical to the 5′ flanking region of the *mcy* gene cluster of *P. rubescens* NIVA-CYA98 (AM990462, Rounge *et al.*, 2009) (Supplementary Figure S1). A pronounced phylogenetic dichotomy indicated that all *mcy*T genes from strains that lost the *mcy* gene cluster were found in one branch (Figure 1a).

**Figure 2:**
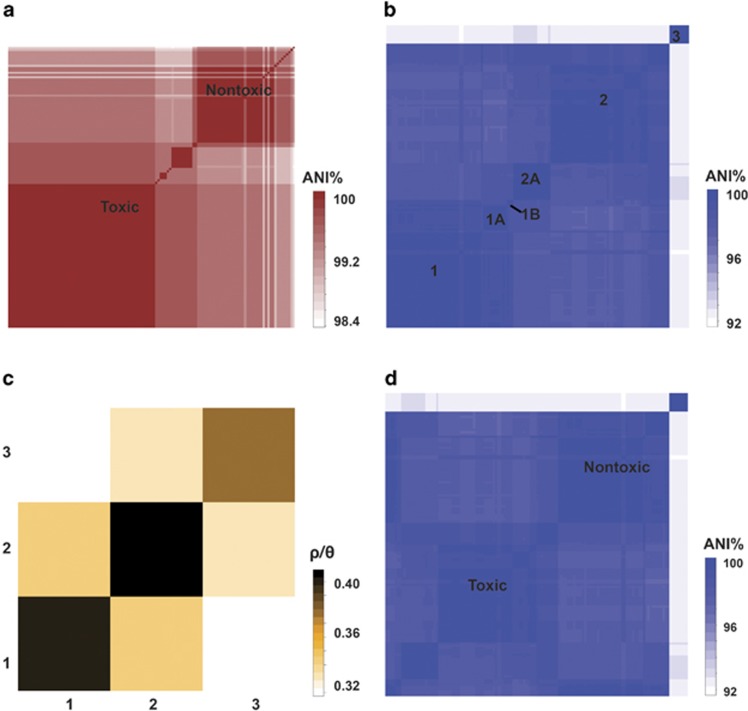
Nucleotide identity of pairwise strain comparisons of (**a**) *mcy*T remnants between strains of different lineages, (**b**) seven housekeeping gene loci between strains of different lineages and (**d**) between strains that retained or lost the *mcy* gene cluster; (**c**) relative rates of recombination and mutation (*ρ*/*θ*) that were calculated between and within each lineage, ANI, average nucleotide identity.

Among the *mcy*T gene, a higher genetic variation was recorded from strains that lost the *mcy* gene cluster (1.25%, 398 bp) compared with 82 strains containing the full *mcy* gene cluster (0.5%, 398 bp; Figure 2a and Table 2). One polymorphism was found (bp 44 A/G) that correlated perfectly with the absence/presence of the *mcy* gene cluster. Interestingly, 19 strains (from Europe and North America) that contain the full *mcy* gene cluster but were found inactive in MC synthesis (Figure 1; Kurmayer *et al.*, 2004) could not be differentiated by nucleotide polymorphism within the *mcy*T gene.

### Identification of phylogenetic lineages and taxonomy assignment

Phylogenetic analyses of 7 housekeeping gene loci among the 138 strains revealed 3 major branches (Figure 1b). The *mcy* genotypes correlated well with phylogeny: one branch of strains that lost or retained the *mcy* gene cluster (lineage 1), one branch of strains exclusively retaining the *mcy* gene cluster (lineage 2), and one branch of strains that exclusively lost the *mcy* gene cluster (lineage 3). The sublineages, 1A, 1B and 2A, were robustly supported; while 1A exclusively consisted of strains that lost the *mcy* gene cluster, 1B and 2A comprised only strains retaining the *mcy* gene cluster. Lineages 1 and 2 consisted of strains occurring across Europe or North America, while lineage 3 consisted of strains from tropical origin. Using MLST, 61 unique STs) were detected, with 37 STs occurring only once. The following clonal complexes were observed: (i) containing only strains of lineage 1 (*n*=42) and 1A (*n*=9), (ii) containing only strains of lineage 2 (*n*=53), and (iii) and (iv) representing two clonal complexes containing only strains of lineage 2A (*n*=4, *n*=10), (Supplementary Figure S2). Although some STs (*n*=13) could not be assigned to the clonal complexes, the branching pattern observed using MLSA was confirmed by MLST as an independent technique.

The average nucleotide identity was >99% within clusters and 92–98% between clusters (Figure 2b). Bayesian analysis using ClonalFrame showed low gene recombination, including HGT related to mutation (*ρ*/*θ*<1) across all clusters (Figure 2c). Furthermore, gene recombination related to mutation between clusters decreased compared with inside the clusters, which suggests low HGT of more distantly related genes. Nucleotide variation among strains that lost the *mcy* gene cluster (average±s.e., 8±1.3%) was higher compared with strains containing the complete *mcy* gene cluster (3.4±2.3% Figure 2d and Table 3). The strains containing the complete *mcy* gene cluster but inactive in MC synthesis (Kurmayer *et al.*, 2004) did not differ in their nucleotide variation compared with the MC-producing strains (2.7±1%). Non-polarized McDonald–Kreitman tests revealed null selective pressure on strains that lost or retained the *mcy* gene cluster within or between each lineage.

Based on housekeeping gene sequences, strains of lineages 1 and 2 could be attributed to *P. agardhii/rubescens*, while the strains among the tropical lineage 3 were assigned to another species *P. pseudagardhii* (Supplementary Table S1) as defined by (Suda *et al.*, 2002). However, the dissimilarity of the 16S rDNA sequence (1361 bp) between *P. pseudagardhii* and *P. agardhii/rubescens* (3.2–4.5%) was higher than previously reported (<1.6%, 63 strains, Suda *et al.*, 2002).

### Pigmentation and adaptation to deep mixing

PC/PE ratios did not change within the observation period of 6 months. The red-pigmented strains from lineage 1A emerged from green-pigmented lineage 1 and had a significantly higher PC/PE ratio when compared with red-pigmented strains from lineage 2 (Kruskal–Wallis one-way analysis of variance on ranks, *P*<0.001, Supplementary Figure S3). Analogously, within all the green-pigmented strains, variable PC/PE ratios occurred, for example, the strains of green-pigmented lineage 2A contained significantly higher amounts of PC compared with the green-pigmented strains of lineages 1, 1A and 2. Nevertheless, the frequency of the pigmentation type varied significantly between lineages, for example, while lineage 1 (49 of 58, 84.5%) and 3 (7 of 7, 100%) were dominated by the green pigmentation type, lineage 2 had a higher proportion of the red pigmentation type (41 of 73, 56.2%, Figure 1).

Similarly to pigmentation, the frequency of *gvp*C gasvesicle protein size genotypes differed between lineages (Figure 1). *Planktothrix* strains assigned to nontoxic lineages 1 and 1A almost exclusively contained the *gvp*C^28^ genotype (49 of 51, 96%). In contrast, strains assigned to lineage 2 frequently contained the *gvp*C^20^ genotype and occasionally the *gvp*C^16^ genotype. The mean (±s.e.) depth of the original habitats of lineages 1 (9±2 m), 1A (6±0) and 3 (8±0) was significantly lower when compared with the depth of the sites of lineages 1B (44±2) and 2 (14±2). The depths of the collection sites of lineage 2A were intermediate (9±1), (Kruskal–Wallis one-way analysis of variance on ranks, *P*<0.001). Using multiple regression analysis, the variables PC/PE pigment ratio (*x*_1_) and mean depth (*x*_2,_ in m, Supplementary Table S1) were included in the forward stepwise method, explaining the *gvp*C genotype frequency: *y*=23.554+2.166*x*_1_−0.105*x*_2_ (adjusted *R*^2^=0.39, *n*=145, *P*<0.001), where *y* is the genetically encoded protein size of *Gvp*C (in kDa). Corresponding results were obtained when using only one strain per ST.

### Peptide composition in strains

The lineages differed significantly in the average number of oligopeptides per strain: Lineage 1 had the lowest number of peptides: 4 (25% percentile)–5 (median)–7 (75% percentile), while lineage 1A (8.3–15–17.8) and lineage 2A (8–10–10) had the highest number (Kruskal–Wallis one-way analysis of variance, *P*<0.001). Lineage 3 only contained unknown peptides. The same trend was obtained when omitting the 64 peptides occurring only once (159 peptides occurred in total).

The peptides (*n*=159) were attributed to eight groups (Table 4): (I) putative sulfate-containing aeruginosins, (II) putative chloride- and sulfate-containing aeruginosins, (III) putative chloride-containing aeruginosins, (IV) MCs, (V) anabaenopeptins, (VI) putative sulfate-containing cyanopeptolins with water molecule fragment, (VII) putative cyanopeptolins with water molecule fragment only, and (VIII) planktocyclins. In general, anabaenopeptins (*n*=100), putative cyanopeptolins (groups VI, VII; *n*=102) and MCs (*n*=64) occurred most frequently and in all the lineages. Planktocyclins occurred with two variants and were the least abundant (*n*=23). The relative frequency of peptides of the eight peptide groups differed significantly between lineages (chi-square test, *χ*^2^ (d.f.=1), *P*<0.001, Supplementary Figure S4).

**Figure 3:**
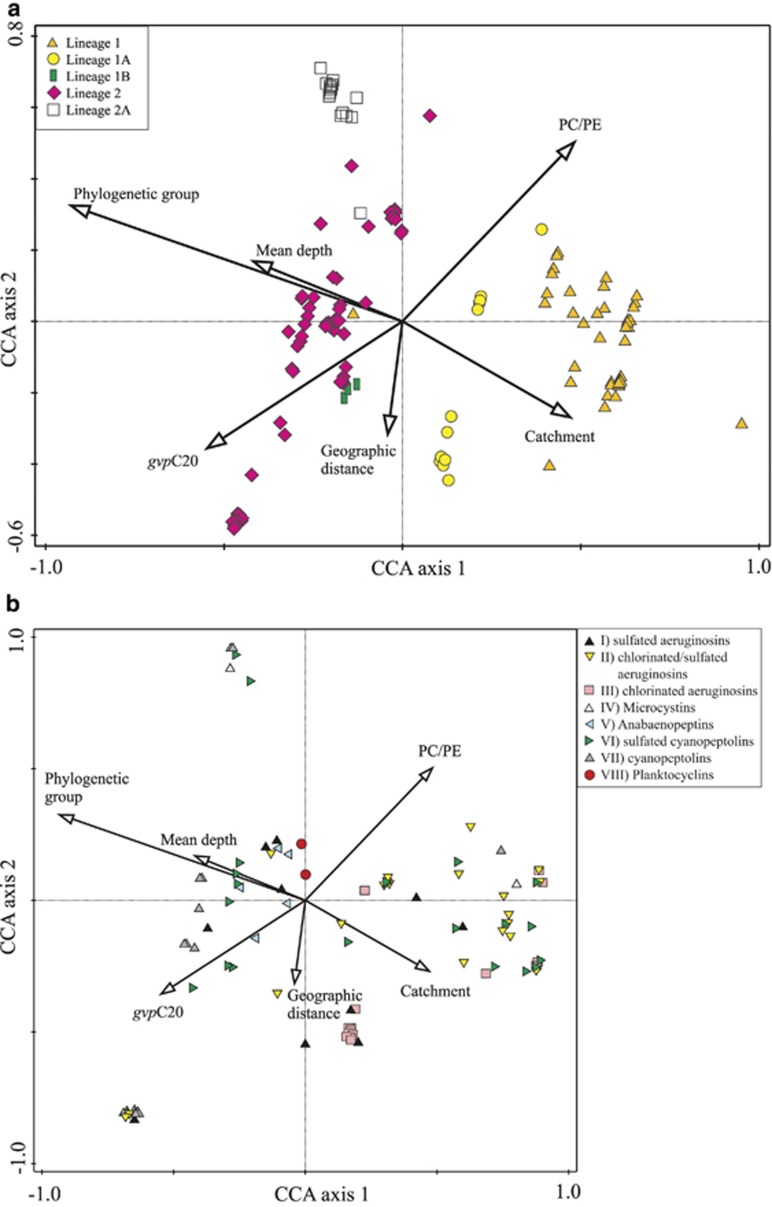
CCA of bioactive oligopeptide occurrence (*n*=95) recorded among 127 *Planktothrix* strains in dependence on phylogeny, ecophysiological adaptations and geographic distance. (**a**) Strains vs parameters where orange triangle=lineage 1, yellow circle=lineage 1A, green box=lineage 1B, pink diamond=lineage 2, and white square=lineage 2A; (**b**) plot of peptides vs parameters where black triangle, yellow triangle—inverted, pink square=different aeruginosins (groups I, II, III), white triangle=MCs (IV), blue triangle—pointed left=anabaenopeptins (V), green triangle—pointed right, grey triangle= different cyanopeptolins (VI, VII), red circle=planktocyclins (VIII); peptides that occurred only once were omitted.

Through the forward selection procedure of direct gradient analysis (CCA), the variables phylogenetic lineage, PC/PE ratio, *gvp*C^20^, geographic distance, mean depth and catchment were included for separating peptide occurrence (*P*<0.05; Figure 3). Corresponding results were obtained when using only one strain per ST (*n*=60, Supplementary Figure S5).

**Figure 4:**
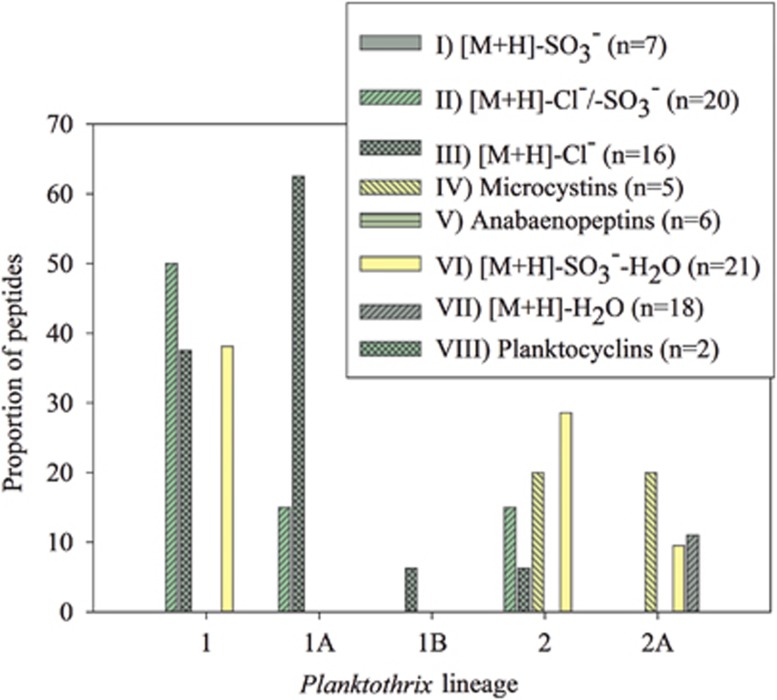
Proportion of peptides for each group that show maximum occurrence within a specific lineage as revealed by CCA (Supplementary Table S1). Lineage 3 did not contain any known oligopeptide.

**Figure 5:**
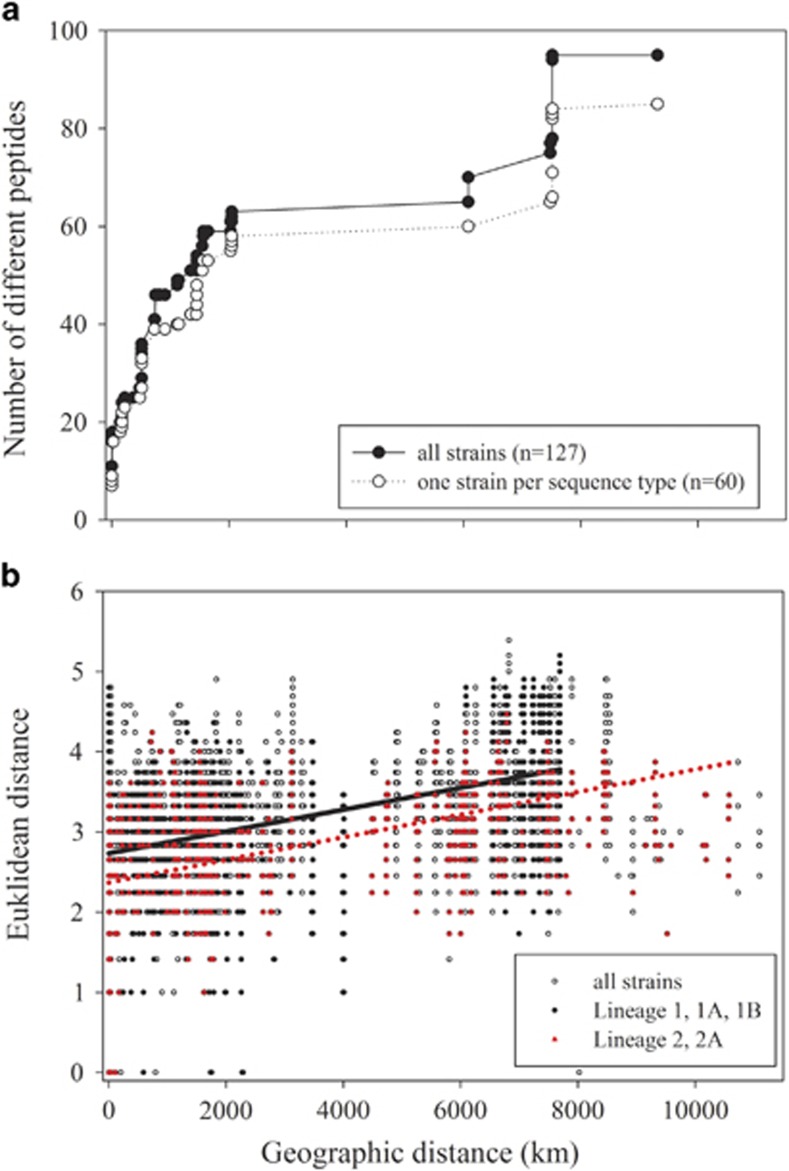
(**a**) Rarefaction curves showing the number of oligopeptides as a function of geographic distance. (**b**) Euclidean distance calculated from the presence/absence of peptides plotted against the geographic distance for each pair of strains (*n*=127). Regression curve details: lineage 1 (1A, 1B), straight line: *y*=2.7328+0.0001*x* (*R*^2^=0.22, *P*<0.0001); lineage 2 (2A), dotted line: *y*=2.3689+0.0001*x* (*R*^2^=0.22, *P*<0.0001), where *y* is Euclidean distance calculated from the presence/absence of peptides and *x* is geographic distance (in km).

None of the peptide groups was explained by a single phylogenetic, physiological or geographic factor. However, CCA revealed a distinct optimum of several peptides of different groups in specific phylogenetic lineages: among groups II, III, VI and VII, many peptides had their maximum occurrence within strains assigned to specific phylogenetic lineages 1, 1A and 2, while lineage 2A had rather few peptides showing maximum frequency (Figure 4; Supplementary Table S2). The geographic distance also had a significant role in peptide occurrence among the strains. For both matrix data sets (strains, *n*=127 and ST, *n*=60), a highly significant correlation between the Euclidean distance of peptide occurrence and geographic distance was found (strains: *n*=127, correlatio*n*=0.36, *t*=7.79, two-tailed *P*=0.001; ST: *n*=60, correlatio*n*=0.26, *t*=3.1, two-tailed *P*=0.002). Moreover, rarefaction curves indicated a linear increase of the peptide numbers up to a geographic distance of 2000 km (Figure 5a), and the linear regression curves indicated that peptide dissimilarity as calculated from Euclidean distance between strains increased significantly as a function of geographic distance (Figure 5b). The slopes of the regression curves of both lineages were similar, suggesting that strains of different lineages did not differ in peptide dissimilarity as a function of geographic distance.

## Discussion

### Ecological differentiation among strains

Suda *et al.* (2002) reported distinct ecophysiological preferences in growth at different temperatures, that is, while strains of *P. agardhii/rubescens* grew best at 10 and 20 °C, strains of *P. pseudagardhii* grew best at 20 and 30 °C. Accordingly, most strains assigned to *P. pseudagardhii* have been isolated from warmer areas, such as East Africa (this study), South Africa (Conradie *et al.*, 2008) and sub-tropical to tropical climate in Thailand and China (Lin *et al.*, 2010). In contrast to *P. pseudagardhii*, all other lineages shared strains that were isolated from the temperate region of the Northern Hemisphere. In both lineages 1 and 2, green- and red-pigmented strains occurred. The phylogenetic assignment of red-pigmented strains among the nontoxic lineage 1A (isolated from North America) indicated that the presence of PE is polyphyletic. Correspondingly, the cellular amount of PC/PE significantly differed between lineages, both among red-pigmented and green-pigmented phenotypes (Supplementary Figure S3). Recently, it has been shown by genome sequence comparison that a PE gene cluster has been horizontally transferred and resulted in red pigmentation in a strain that was otherwise more closely related to green-pigmented strains (Tooming-Klunderud *et al.*, 2013). Similar to planktonic unicellular cyanobacteria (Haverkamp *et al.*, 2009), the pigmentation may be more frequently modified in response to prevailing light absorption maxima than previously assumed. Nevertheless, in the field, the co-occurrence of green- and red-pigmented *Planktothrix* strains has been described only occasionally (Davis and Walsby, 2002; Kurmayer *et al.*, 2011), which corresponded to ecological differentiation, for example, in deep-stratified lakes the red-pigmented life form consistently seemed to outcompete the green-pigmented life form (Davis *et al.*, 2003).

Strains of nontoxic lineages 1 and 1A almost exclusively contained *gvp*C^28^ encoding gas vesicles with a relatively wide diameter (28 kDa). Strains of toxic lineage 2 typically contained *gvp*C^28^ and *gvp*C^20^, while some strains of lineage 2 also contained *gvp*C^16^, which is known to resist high hydrostatic pressure because the encoded gas vesicle protein has the smallest diameter (16 kDa; Beard *et al.*, 2000). Among the phylum of cyanobacteria (including *Dactylococcopsis*, *Aphanizomenon*, *Microcystis* and green- and red-pigmented *Oscillatoria* from deep lakes), a significant negative relationship between gas vesicle protein pressure resistance and gas vesicle diameter has been reported (Walsby and Bleything, 1988). Within *Planktothrix*, it could be demonstrated that the size of the gas vesicle protein shows a negative correlation with the resistance to the critical pressure required to collapse the gas vesicle (Bright and Walsby, 1999). As nonbuoyant filaments are less likely to contribute to population growth during the next season, selective pressure would favor those genotypes that can maintain their buoyancy even during holomixis (Beard *et al.*, 2000). The deep lake habitats sampled during this study typically are dimictic and are mixed to their greatest depth during spring and autumn (Posch *et al.*, 2012). Thus we propose that genotypes of lineage 2 constitute an ecotype containing gvpC^20^ and gvpC^16^ that is adapted to deep-mixing events occurring regularly in deep lakes such as those of the Alps. In contrast, the genotypes of lineages 1, 1A and 2A constitute an ecotype containing gvpC^28^ typically occurring in the shallow water bodies lacking the selective pressure of deep mixing.

### Origin of nontoxic strains

Overall, the presence of the *mcy* gene cluster showed a clonal dependence resulting in a correlation between phylogeny and *mcy* gene cluster distribution. Accordingly, the nucleotide diversity within the *mcy*T gene not only correlated with the presence of the *mcy* gene cluster but also with its phylogenetic assignment. We conclude that the nucleotide polymorphism within the *mcy*T gene could be used to differentiate strains that lost the *mcy* gene cluster from those still containing the full *mcy* gene cluster. The only exception we know so far was found in strain No. 252 that does not contain any remaining *mcy*T but the 5′ end flanking region of the *mcy* gene cluster as well as the *mcy*J gene located at the 3′ end of the *mcy* gene cluster (see Figure 3, type IV of gene cluster deletion event, Christiansen *et al.*, 2008a). Nevertheless, as No. 252 shared identical insertion element residues with all the other nontoxic strains, common ancestry was concluded. Surprisingly, even among the strains assigned to *P. pseudagardhii*, evidence of *mcy* gene cluster loss was found, as those remnants contained the 5′ end flanking region and part of the *mcy*T gene. It is concluded that the *mcy* gene cluster was lost before the speciation event of *P. agardhii/rubescens* and *P. pseudagardhii* (Supplementary Figure S6).

The highest similarity of the *mcy* gene cluster remnants crossing species boundary (between *P. agardhii*/*rubescens* and *P. pseudagardhii*) is intriguing taking into account the relatively high dissimilarity between the species within 16S rDNA (see above). The best explanation for this highest similarity of *mcy* gene cluster remainders across species is that the probability of point mutations within *mcy* gene remnants is significantly lower than within 16S rDNA. In contrast to earlier assumptions, 16S rDNA does not show a fixed rate of evolution when compared with other genes in the genome but evolves either slower or at a faster rate (Kuo and Ochman, 2009). Nevertheless, the observed genetic variation within 16S rDNA among nontoxic *Planktothrix* spp. strains in our study confirmed the conclusion that *mcy* gene loss happened a relatively long time ago, that is, several millions of years from now (Christiansen *et al.*, 2008a). It is hypothesized that (i) an ancestral *Planktothrix* genotype lost the *mcy* gene cluster and (ii) subsequently both toxic and nontoxic genotypes co-existed forming a lineage 1 (1A, 1B) for evolutionary relevant periods. By contrast, lineage 3 (*P. pseudagardhii*) emerged from a nontoxic genotype that became adapted to the (sub)tropical climate. The toxic lineage 2 (2A) originated more recently and frequently contained red-pigmented strains assigned to *P. rubescens*. Indeed, DNA–DNA hybridization experiments suggested that *P. rubescens* originated from *P. agardhii* relatively recently (Suda *et al.*, 2002). In summary, the ecological diversification of either a genotype that lost or a genotype that retained the *mcy* gene cluster can explain the contrasting proportion of *mcy* genes in populations growing in individual habitats (Kurmayer *et al.*, 2011). During a 29-year observation period of deep Lake Zürich (max. depth=136 m), it was found that strains of lineage 1 containing nontoxic strains always were present but never became dominant (Ostermaier *et al.*, 2012). Thus, by strain isolation, from deep lakes in the Alps typically only strains containing the full *mcy* gene cluster have been recorded while from shallow lakes both toxic and nontoxic strains have been isolated (Supplementary Table S1).

### Peptide structural variation in dependence on phylogenetic and geographic distance

Phylogeny had the strongest influence on peptide distribution among strains: the frequency of occurrence of chlorinated and sulfated aeruginosins (group II) and cyanopeptolins (group VI) was high within the strains of lineage 1 (1A; Figures 3 and 4). In contrast, the frequency of occurrence of MCs (IV), anabaenopeptins (V) and cyanopeptolins (VII) rather correlated with the strains assigned to lineage 2, 2A. Specific chlorinated and sulfated aeruginosins also occurred among the strains of lineage 2. Cadel-Six *et al.* (2008) investigated 28 *Microcystis* strains for the presence of chlorinated aeruginosins or cyanopeptolins and the functional integration of the putative halogenase *aer*J or *mcn*D in the corresponding nonribosomal peptide synthetases. The same authors found short direct repetitive sequences flanking the *aer*J or *mcn*D genes that might favor HGT. Therefore it might be possible that modificatory genes such as *aer*J or *mcn*D not only have been acquired during the evolution of lineage 1 (1A) but also have been transferred sporadically to individual genotypes assigned to lineage 2.

Another factor that contributed to peptide structural diversification was geographic distance; for example, in lineage 1A all strains isolated from a single habitat (Moose Lake, Alberta, CA, USA) contained chlorinated aeruginosins, whereas strain No. 277 in the same lineage but isolated from Wannsee (Germany) did not contain chlorinated peptides. In other words, the dissimilarity of peptide structural variation within the genus *Planktothrix* increases with geographic distance (Figure 5). It is known that spatial isolation can result in the occurrence of rare and previously unknown MC structural variants (Kurmayer *et al.*, 2004). Although structural variation could be linked to microevolutionary changes in the respective nonribosomal peptide synthetases genes (Christiansen *et al.*, 2008b), it also could be shown that even closely located *Planktothrix* populations in lakes of the Alps were sufficiently isolated that rare genotypes could develop and dominate populations (Kurmayer and Gumpenberger, 2006). Following the theory of overall selective neutrality in secondary metabolite structural variation, for example, Firn and Jones (2000), a major part of the variability in MC structural variants could be explained by random drift (Kurmayer and Gumpenberger, 2006). Extrapolating this conclusion to other oligopeptide groups would imply that random drift also accounts for peptide structural variation between spatially separated populations. Consequently, we think it is both microevolutionary changes and random drift that contribute to the observed peptide dissimilarity vs geographic distance relationship. Indeed, ‘founder effects' have been invoked repeatedly to explain the spatial and even biogeographic differences among microorganisms (De Meester, 2011).

### Functional replacement of MC

It is tempting to assume that the loss of toxicity of MC might be replaced by other bioactive peptides, such as oscillapeptin or planktocyclin, that have been shown to deter potential grazers (see above). Recently, Kohler *et al.* (2014) described the chlorinated and sulfated aeruginosin 828A isolated from nontoxic strain No. 91/1 that inhibited the serine proteases thrombin and trypsin in the low nanomolar range. Other MC-deficient strains (Nos. 405, 406, 496/1, 550, 551) also contained chlorinated/sulfated aeruginosins of unknown toxicity. However, aeruginosin 828A also occurred in strains still producing MC, which were all assigned to lineage 2. Therefore, a hypothetical HGT of *aer*J or *mcn*D and their subsequent functional integration into the respective gene clusters already occurred under MC-producing conditions. In summary, no clear evidence for an increased abundance of certain peptides in the absence of MC was found. The facultative loss of MC seems to be compensated by a range of different oligopeptides derived from different peptide families rather than a certain peptide structure.

## Conclusion

We provide evidence that the *mcy* gene cluster was lost before *Planktothrix* speciation events occurred, that is, before the speciation between *P. agardhii*/*P. rubescens* and *P. pseudagardhii* and between *P. rubescens* and *P. agardhii*. The observed *mcy* gene cluster remnant might be considered a pseudogene and might be used to estimate the timescale of *mcy* gene cluster loss events both within and across species and genera. This timescale may be further linked to the presence of additional bioactive peptide synthesis pathways in order to understand the evolution of secondary metabolite synthesis in general.

## Figures and Tables

**Table 1:** Summary on characteristics of *Planktothrix* strains used in this study and grouped according to the phylogenetic lineages as shown in Figure 1b

*Phylogenetic group*	*Number of strains*	*Number of STs*	*Number of sampled waterbodies*	*Number of sampled countries*	*mcy gene cluster present*	*GvpC (gas vesicle protein size in kDa)*	*Pigmentation*	*PC/PE ratio*	*Mean depth*	*Maximum depth*	*Area km*^*2*^	*Catchment km*^*2*^	*Latitude (°)*	*Longitude (°)*	*Distance to Mondsee (in km)*
1	44	21	16	11	No/yes	28	G^a^	1.5–1.95–2.26	1–9.5–37	2–15.6–43	0.1–23–396	0.1–1793–7000	39.2–48.7–60	111–3–39	178–1515–7572
1A	11	6	2	2	No	28	G/R	0.4–1.2–2	5.6–5.6–6	9–19–20	2.8–37–41	755–1323–7000	52–54–54	–111–99–13	519–6931–7572
1B	3	2	2	2	Yes	20	R	0.26–0.28–0.3	42–44–47	81–84–86	19–29–47	162–439–994	46–47–47	11–13–14	159–167–183
2	56	23	21	9	Yes	28;20;16	R/G	0.17–0.7–2.4	4–13–52	9–34–136	0.07–12–220	0.1–293–7000	37.6–47.1–59.5	110–19–16.2	0–2439–7531
2A	17	8	4	2	Yes	28	G	2–2.3–2.6	6–8–23	21–28–64	1–12–30	16–308–935	59.5–60–61	10.5–23–25	1337–1575–1598
3	7	1	1	1	No^b^	28;20;16	G	2–2.2–2.5	3.6	8	1.4	10	0.4	30.2	5468

Abbreviations: PC, phycocyanin; PE, phycoerythrin; ST, sequence type.

For each strain, an ST was defined from seven sequenced gene loci according to Multi Locus Sequence Typing (Feil *et al.*, 2004). For strain-specific details, see Supplementary Table S1. Data are presented as min–mean–max.

a G, green-pigmented; R, red-pigmented.

b Smallest remnant of *mcy*T and the presence of the 5′ flanking region of the *mcy* gene cluster (Supplementary Figure S1).

**Table 2:** Sequence variation within the *mcy*T gene that is part of the *mcy* synthesis gene cluster

*Group*	N *strains*	*bp*	*Max. dissimilarity (%)*	N *variable sites*	N *alleles*
All strains	124^a^	398	1.5	24	23
Strains containing the *mcy* gene cluster	82	398	0.5	8	9
Subgroup active in MC synthesis	6263	398	0.5	6	7
Subgroup inactive in MC synthesis	19	398	0.5	2	3
Strains lacking the *mcy* gene cluster	42^a^	398	1.25	17	14
Subgroup *mcy* 5′ end flanking region and reduced *mcy*T	14^a^	443	1.58	5	6
Subgroup *mcy* 5′ end flanking region	1^b^	193	—	—	—

Abbreviation: MC, microcystin.

a Strain No. 713 had a 202-bp insertion that was identical to the 5′ flanking region of the *mcy* gene cluster of *P. rubescens* NIVA-CYA98 (AM990462, Rounge *et al.*, 2009) (Supplementary Figure S1).

b Strain No. 252 only showed the 5′ end flanking region of the *mcy* gene cluster in *P. agardhii* toxic strain NIVA-CYA126/8 but *mcy*J gene and the 3′ end flanking region (Christiansen *et al.*, 2008a).

**Table 3:** Sequence variation of the seven loci used for MLSA in *Planktothrix* strains

*Locus*	*bp*	N	*Max. dissimilarity (%)*	*Number of variable sites*	*Number of alleles*
*All strains*					
16S rRNA	301	138	1.99	7	4
16S-ITS	312	138	8.2	32	8
PC-IGS	204	138	12.8	37	13
PSA-IGS	548	138	8.8	55	7
RNaseP	299	138	7.5	28(+56)^a^	13
*rbc*LX	336	138	5.9	42	7
*rpo*C	492	138	11.6	73	14
Average (±s.e.)			8.1±3.6^b^	39±8^b^	9.4±1.5^b^
					
*Strains containing the mcy gene cluster*					
16S rRNA	301	82	0	0	1
16S-ITS	312	82	2.6	11	3
PC-IGS	204	82	6.9	20	11
PSA-IGS	548	82	3.5	20	5
RNaseP	241	82	1.7	8	8
*rbc*LX	336	82	4.3	31	6
*rpo*C	492	82	5.1	31	9
Average (±s.e.)			3.4±2.3^c^	17±4b,c	6.1±1.3b,c
					
*Subgroup active in MC synthesis*					
16S rRNA	301	63	0	0	1
16S-ITS	312	63	2.3	10	2
PC-IGS	204	63	6.9	20	10
PSA-IGS	548	63	3.5	20	5
RNaseP	241	63	1.2	7	5
*rbc*LX	336	63	3.7	27	5
*rpo*C	492	63	4.9	31	8
Average (±s.e.)			3.2±2.3^c^	16±4b,c	5.1±1.2b,c
					
*Subgroup inactive in MC synthesis*					
16S rRNA	301	19	0	0	1
16S-ITS	312	19	0.3	1	2
PC-IGS	203	19	6.9	14	3
PSA-IGS	548	19	3.3	18	3
RNaseP	241	19	1.2	3	3
*rbc*LX	336	19	1.8	13	3
*rpo*C	492	19	5.1	27	4
Average (±s.e.)			2.7±2.6^c^	11±4^c^	2.7±0.4^c^
					
*Strains lacking the mcy gene cluster*					
16S rRNA	301	56	1.99	7	4
16S-ITS	312	56	8.2	32	6
PC-IGS	204	56	12.8	30	5
PSA-IGS	548	56	8.8	48	4
RNaseP	299	56	7.5	23(+56)^a^	7
*rbc*LX	336	56	5.9	34	4
*rpo*C	492	56	11	60	6
Average (±s.e.)			8.0±3.5^b^	33±6b,c	5.1±0.5b,c

Abbreviations: IGS, intergenic spacer region; MC, microcystin; MLSA, Multi Locus Sequence Analysis; PC, phycocyanin.

a (Plus insertions).

b,c Identical superscripts mark groups that are not significantly different at *P*<0.05 (Tukey's *post-hoc* test).

**Table 4:** Peptide groups as identified from all strains (*n*=134)^a^ by LC–MS and the proportion of strains containing a specific peptide group according to phylogenetic lineages identified in Figure 1b

*Peptide group*	*M+H*	*No. of peptides*	*Total frequency of occurrence (%)*	*Proportion (%) of strains containing a specific peptide group*					
				*Lineage 1 (*n*=40)*	*Lineage 1A (*n*=11)*	*Lineage 1B (*n*=3)*	*Lineage 2 (*n*=56)*	*Lineage 2A (*n*=17)*	*Lineage 3 (*n*=7)*
(I) Sulfated aeruginosins (-SO_3_^−^)	639–845	16	39 (29%)	23	64	0	39	6	0
(II) Chlorinated/sulfated aeruginosins (-Cl^−^/-SO_3_^−^)	687–893	29	51 (38%)	73	27	0	34	0	0
(III) Chlorinated aeruginosins (-Cl^−^)	599–1165	25	27 (20%)	43	64	33	4	0	0
(IV) Microcystins	887–1046	5	64 (48%)	15	0	67	71	94	0
(V) Anabaenopeptins	810–859	6	100 (75%)	38	100	100	96	100	0
(VI) Sulfated cyanopeptolins (-SO_3_^−^-H_2_O)	947–1171	44	66 (49%)	78	82	100	32	29	0
(VII) Cyanopeptolins (-H_2_O)	831–1193	32	36 (27%)	5	9	0	38	71	0
(VIII) Planktocyclins	802–817	2	23 (17%)	13	0	33	30	0	0
Total		159							

Abbreviation: LC–MS, liquid chromatography–mass spectrometry.

a Four strains went extinct before the peptide analysis was performed.
