# Supplementary material for: Integrating phylogeny, geographic niche partitioning and secondary metabolite synthesis in bloom-forming Planktothrix
Source: ISME J. 2014 Oct 17;9(4):909–21. doi: 10.1038/ismej.2014.189 (PMC4349496; doi:10.1038/ismej.2014.189)
Supplement: Supplementary Information [file ismej2014189x1.doc]

**Supporting Information**

***Full Methods***

**Organisms**

All clonal *Planktothrix* strains were isolated by cutting out single filaments migrating on agar as described , and cultivated at 15°C under low light conditions (5-10 µmol m^-2^ s^-1^; Osram Type L30W/77 Fluora) in BG11 medium . Regular transfer was carried out every six weeks to fresh medium. Cells were frozen at -20°C, then harvested by centrifugation (14 000 g) for DNA extraction following standard procedures. In total 138 strains were analyzed, including 62 isolated previously ; Table 1, Suppl. Table 1).

**Genetic analysis of strains**

*Planktothrix* strains were analyzed for (i) the presence of the *mcy*T gene, which occurred in toxic strains and as a remainder in nontoxic strains, (ii) the genetic variation within seven housekeeping gene loci and intergenic spacer regions (IGS): 16S rDNA (301 bp), 16S rDNA-ITS (305 bp), phycocyanin (PC)-IGS (203 bp), PSA-IGS (IGS between photosystem I related *psa*A and *psa*B, 548 bp), RNaseP (241 bp), *rbc*LX-IGS (IGS between the large subunit of the ribulose bisphosphate carboxylase/oxygenase and *rbc*X, 336 bp), *rpoC* (492 bp) using multi locus sequence typing or MLST . In addition, the ability to express gas vesicle protein variants of 28, 20, 16 kDa was tested using a diagnostic PCR protocol using primers (*gvp*C9, *gvp*C1B) to reveal the presence of genes *gvp*C^28^ (621 bp), *gvp*C^20^ (402 bp), *gvp*C^16^ (303 bp) according to previous description .

The presence of the full *mcy* gene cluster (*mcy*A-T, AJ441056, Christiansen *et al.* 2003) was checked via PCR amplification of transition regions between the *mcy* genes as described previously (Kurmayer et al. 2004). The presence of the *mcy*T gene in 82 toxic and 36 nontoxic strains was tested using the *mcy*TA-Z+ and *mcy*TA-Z- primers revealing a product of 791 bp under standard PCR conditions as reported previously (Christiansen *et al.*, 2008). Six nontoxic strains (No394, 757, 771, 776, 816, 867) did not contain the whole *mcy*T gene but only a part of it which was amplified using the primers 474+: 5’-AATAGCGATTTTCCCAAGCATTC-3’ and 474-: 5’-TGGTCACAGTATGGGCGGAT-3’ (451 bp). Another 14 nontoxic strains (No252, 354, 390, 704-713, 759, 782, 787, 826) only contained a smallest part of the *mcy*T gene that still could be amplified by primers spanning the 5’ region of the *P. agardhii* *mcy* gene cluster (Access No AJ441056): *mcy*T_lifwd: 5’-AAATTGTGCCATTCTCAATTGA-3’ and *mcy*T_lirev 5’-GGTGGATTAGAAGATTTGAAACCTAA-3’ (506 bp).

The oligonucleotides for amplification of the 16S rDNA, 16S rDNA-ITS, PC-IGS, and PSA-IGS have been reported previously (Christiansen *et al.*, 2008). The oligonucleotides to amplify RNaseP were re-designed from previously described using sequences of *Prochlorococcus*, *Calothrix*, *Anabaena*, *Synechocystis* and *Planktothrix* (Access. No. AJ272220, AJ272221, AJ272222, X65649, X65648, X65707): RnasePfwd: 5’-CTGAGGAAAGTCCGGGCT-3’ and RnasePrev: 5’-AACAGAACCCGGCTTATGTC-3’ revealing a PCR product of 312 bp. The oligonucleotides used to amplify the *rbc*LX locus were designed from *Planktothrix* sequences available from Genbank : AY583373 - AY583379; : Z94866 - Z94875): *rbc*fwd 5’- TGTTCAAGCGCGTAACGAAG – 3’ and *rbc*rev 5’ - CCAGGGTTAGTTTCCCATAGTTGAT – 3’ (382 bp). Similarly, the oligonucleotides used to amplify *rpo*C1 were designed from sequences from Genbank from *Planktothrix* (EU151913, AY425002-3, : *rpo*F 5’ - TGGTCAAGTGGTTGGAG -3’ and *rpo*R 5’- GCCGTAAATCGGGAGGAA – 3’(678 bp).

PCR was performed using Dream Taq Polymerase (Thermo Scientific, St. Leon Rot, Germany) following manufacturer’s instructions. The temperature during the annealing step varied from 55-60°C (16S rRNA: 60°C; 16S-ITS rRNA: 60°C; PC-IGS: 52°C; PSA-IGS: 58°C; RNaseP: 56°C; *rbc*LX: 55°C; *rpo*C: 55°C). PCR products were purified and sequenced either directly or cloned according to standard procedure. The sequences have been submitted to Genbank under the following accession numbers: KC235395 - KC235407 (5’flanking region of the *mcy* gene cluster and *mcy*T remainders with 443 or 646 bp), KC235408 - KC235470 (*mcy*T with 397 or 398 bp), KC235837 - KC235912 (16S ribosomal RNA with 301 bp), KC235761 - KC235836 (16S-23S ribosomal RNA-ITS with 305 or 307 or 308 or 309 bp), KC235685 - KC235760 (PC-IGS with 202-203 bp), KC235609 - KC235684 (PSA-IGS with 548 bp), KC235471 - KC235608 (RNaseP with 241 or 297 bp), KC236051 - KC236188 (*rbc*LX with 321 or 329 or 336 bp), KC235913 - KC236050 (*rpo*C with 492 bp).

**Pigment analysis of strains**

For pigment analysis (phycocyanin/phycoerythrin or PC/PE ratios) frozen cells were analyzed for water soluble phycobilins according to previous description . Each strain was analyzed in duplicate within a time interval of six months.

**Oligopeptide analysis of strains**

For MC extraction a minimum of 2 mg of dry weight was harvested onto glass fibre filters and extracted in aqueous methanol as described . All strains were tested for MC production by the same two independent methods: with the protein phosphatase 1A inhibition assay and via HPLC-DAD .

For general oligopeptides analysis, freeze dried biomass of all strains (median 13.9 mg of dry weight) was extracted for oligopeptides in 50% (v/v) aqueous methanol (4.5 ml) on ice . The separation of peptides was performed on a Shimadzu 10AVP system equipped with a photodiode array detector on an analytical Hydrosphere C18 reversed phase column (4.6 × 250 mm; 5 µm particle size, 12 nm pore size; YMC Europe GmbH, Dinslaken, Germany). The flow rate was 1 ml min^-1^ and oven temperature was set at 30°C. Two different solvents were used, solvent A was composed of UV-treated deionized H_2_O and 0.05% trifluoroacetic acid (TFA) and solvent B was HPLC-grade acetonitrile and 0.05% TFA. A linear increase in two steps was applied (solvent B from 20 to 70% in 50 min, 70 to 100% in 2 min, isocratic 8 min). The injection volume was 100 to 200 µl. The HPLC system was directly coupled to an Electrospray Ionisation (ESI) Mass Spectrometer (MS; LCQ Duo ion trap mass spectrometer, Thermoquest, Finnigan) operating in positive ion mode. Both, the sheath gas and the auxiliary gas were nitrogen and set to 75 and 20 arbitrary units, respectively. The capillary temperature was 200°C. Capillary voltage and spray voltage were set to 26 V and 4.5 kV, respectively. All data were recorded in full scan mode ranging from *m/z* 200-2000 atomic mass units (amu). The maximum ionization time was set to 200 ms, three microscans were combined for each data point. For each strain a peptide fingerprint profile was obtained. For the families of the MCs, anabaenopeptins, planktocyclins all peptides could be identified according to our own previous work or the literature . For aeruginosins and cyanopeptolins the molecular mass of already elucidated oligopeptides was used to identify the peptide family as well as the individual peptide (SciFinder; Chemical Abstracts Service: Columbus, OH, 2014; Suppl. Table 2).

**Phylogenetic analysis**

Sequences of partial *mcy*T gene (398 bp, n = 124) were aligned using multiple sequence alignment (Clustal W 1.8), and nucleotide substitution parameters were estimated by Maximum likelihood analysis (BASEML of the PAML package, under a general time-reversible nucleotide substitution model by estimating the gamma distribution for variable rates among the sites. Ambiguous sites (at least one sequence showed a gap) were removed (three sites) and the discrete gamma algorithm was used to approximate a continuous gamma distribution using five categories of rates (ncatG = 5): alpha (gamma, K = 5) = 1.68390, Average Ts/Tv = 2.4403).

The sequences of the 16S rDNA, 16S rDNA-ITS, PC-IGS, PSA-IGS, RNaseP, *rbc*LX-IGS, and *rpo*C were concatenated resulting in 2,697 bp. Ambiguous sites (n = 93) were removed from the sequence alignment when approximating a continuous gamma distribution (ncatG = 5): alpha (gamma, K = 5) = 0.01712, Average Ts/Tv = 2.5996.

Phylogenetic trees were constructed using (i) maximum likelihood (ML), (ii) neighbour-joining (NJ) from the nucleotide sequences distance matrix (calculated using the F84 substitution model), and (iii) maximum parsimony (MP) from nucleotide sequences using the PHYLIP package . Statistical significance of the branches was estimated by bootstrap analysis generating 1000 replicates of the original data set using the PHYLIP package. Finally, consensus trees following the 50% majority rule were computed.

For multi locus sequence typing (MLST) all 138 strains were defined by the alleles (unique genotypes) present at the seven gene loci (the allelic profile). A nonredundant database (NRDB) tool was used to find those strains that were identical (<http://pubmlst.org/> ). Each unique allelic profile was assigned a sequence type (ST). Isolates with the same ST at all loci were considered to be members of a single clone . The program eBurst (V3) was used at the MLST website (<http://www.mlst.net/> ) to divide the strains into clonal complexes. Each of the clonal complexes contained only strains sharing at least six of seven identical alleles with at least one other strain in the group.

**Recombination analyses.** The Bayesian-based ClonalFrame was used to measure the frequency of recombination within and between different clusters. ClonalFrame predicted clonal genealogy after 5,000 burn-in iterations and 5,000 iterations based on comparing genealogies of triplicate runs. The ratio of rates at which recombination events happen relative to mutations (*ρ/θ*) was caculated.

**McDonald-Kreitman (MK) selection tests.** Non-polarized MK tests were calculated between clusters . The number of synonymous and nonsynonymous substitutions was detected and used to calculate the Neutrality Index (NI) and evaluated using Chi-Square test.

**Statistical analysis of peptide composition**

In order to determine the dependence of peptide occurrence on phylogenetic and environmental parameters (Suppl. Table 1), Canonical Correspondence Analysis (CCA) was performed (CANOCO 5.0 for Windows, . For this purpose two matrices were constructed, (i) one containing the log(x+1) transformed variables (n = 10) describing the phylogeny, ecophysiology (PC/PE ratio, *gvp*C^16^, *gvp*C^20^, *gvp*C^28^ gene presence) and environment (maximum depth, mean depth, area of the waterbody, catchment area, and geographic distance) for each strain (n = 127) or ST (n = 60), and (ii) one containing the presence/absence data on peptide occurrence (n = 95) for each strain (n = 127) or ST (n = 60). Peptides that occurred only once were excluded (n = 64). Multicollinearity of variables was controlled using the variance inflation factor (VIF) and variables showing VIF >2 were a priori excluded (*gvp*C^16^, *gvp*C^28^, max. depth, area of the water body). The forward selection procedure was used and the statistical dependence between the canonical axes, environmental variables and peptide distributions was tested by Monte Carlo permutation tests according to default settings.

Overall, CCA showed a significant relationship between the explanatory variables and peptide composition (p = 0.002). The first and second axis cumulatively explained 28.3% and 51.1% of the peptide composition variability and showed a high correlation with the environmental data (r = 0.85 and r = 0.82).

In order to test the spatial dependence of peptide occurrence an Euclidean distance matrix calculated from peptide presence/absence data for individual strains (n=127)/ MLST (n=60) and a geographic distance matrix for individual strains/ST calculated from sample origin coordinates (Suppl. Table 1) were compared using the Mantel test. For the permutation analysis (1000 permutations) and testing of significance (two-tailed test), the default settings of the PASSaGE softare version 2.0 were used .

**Suppl. Table** **1.** Summary of all *Planktothrix* strains used in this study grouped according to the phylogenetic lineages as shown in Figure 1. For each strain a sequence type (ST) was defined from seven sequenced gene loci according to MLST (Feil *et al*., 2004). Nd, no data. GvpC, size of gas vesicle protein C (in kilo Dalton), Species assignment following Suda *et al.*, (2002): *aga.* stands for *P. agardhii* (Gomont) Anagnostidis & Komárek 1988; *rub.* stands for *P. rubescens* (D.C. ex Gomont) Anagnostidis et Komárek 1988; *psu.* stands for *Planktothrix pseudagardhii* sp. nov. Suda & Watanabe.

| **Phyl. lineage** | **Strain No** | **ST (MLST)** | **Origin** | **Taxon** | ***mcy* gene cluster** | **GvpC variant (kDa)** | **Pigment^4^** | **Ratio 615/562** | **Mean depth** | **Maxium depth** | **Area km^2^** | **Catchment km^2^** | **Latitude(°)** | **Longitude(°)** |
| --- | --- | --- | --- | --- | --- | --- | --- | --- | --- | --- | --- | --- | --- | --- |
| 1 | 31/1 | 11 | Wannsee (DE) | *aga.* | yes | 28 | G | 1.73 | 6 | 9 | 2.8 | 7000 | 52.43 | 13.2 |
| 1 | 32 | 29 | “ | “ | yes | 28 | G | 1.50 | 6 | 9 | 2.8 | 7000 | 52.43 | 13.2 |
| 1 | 39 | 14 | “ | “ | yes | 28 | G | 2.06 | 6 | 9 | 2.8 | 7000 | 52.43 | 13.2 |
| 1 | 41 | 6 | Jägerteich (AT) | “ | no | 28 | G | 1.95 | 1 | 2 | 0.2 | 6.2 | 48.82 | 15.27 |
| 1 | 63 | 37 | “ | “ | no | 28 | G | 2.01 | 1 | 2 | 0.2 | 6.2 | 48.82 | 15.27 |
| 1 | 66 | 6 | “ | “ | no | 28 | G | 2.05 | 1 | 2 | 0.2 | 6.2 | 48.82 | 15.27 |
| 1 | 79 | 22 | L. ArresØ (DK) | “ | yes | 28 | G | 2.10 | 6 | 40 | 5.6 | 216 | 55.97 | 12.1 |
| 1 | 250 | 4 | Albufera Lagune (ES) | “ | no | 28 | G | 2.05 | 1 | 3 | 21 | 917 | 39.34 | -0.36 |
| 1 | 251 | 4 | “ | “ | no | 28 | G | 2.03 | 1 | 3 | 21 | 917 | 39.34 | -0.36 |
| 1 | 252 | 7 | “ | “ | no^2^ | 28 | G | 1.82 | 1 | 3 | 21 | 917 | 39.34 | -0.36 |
| 1 | 253 | 8 | “ | “ | no | 28 | G | 2.01 | 1 | 3 | 21 | 917 | 39.34 | -0.36 |
| 1 | 254 | 15 | “ | “ | no | 28 | G | 2.10 | 1 | 3 | 21 | 917 | 39.34 | -0.36 |
| 1 | 255 | 16 | “ | “ | no | 28 | G | 2.03 | 1 | 3 | 21 | 917 | 39.34 | -0.36 |
| 1 | 256 | 38 | “ | “ | no | 28 | G | 2.0 | 1 | 3 | 21 | 917 | 39.34 | -0.36 |
| 1 | 257 | 9 | “ | “ | no | 28 | G | 1.97 | 1 | 3 | 21 | 917 | 39.34 | -0.36 |
| 1 | 259 | 8 | Wannsee (DE) | “ | no | 28 | G | 1.97 | 6 | 9 | 2.8 | 7000 | 52.43 | 13.2 |
| 1 | 263 | 8 | “ | “ | no | 28 | G | nd | 6 | 9 | 2.8 | 7000 | 52.43 | 13.2 |
| 1 | 274 | 34 | “ | “ | no | 28 | G | 1.91 | 6 | 9 | 2.8 | 7000 | 52.43 | 13.2 |
| 1 | 281 | 8 | “ | “ | no | 28 | G | 1.65 | 6 | 9 | 2.8 | 7000 | 52.43 | 13.2 |
| 1 | 299 | 8 | Klinkenbergerplas (NL) | “ | no | 28 | G | 1.59 | 13.5 | 30 | 0.3 | 1045 | 52.2 | 4.49 |
| 1 | 307 | 8 | “ | “ | no | 28 | G | 1.92 | 13.5 | 30 | 0.3 | 1045 | 52.2 | 4.49 |
| 1 | 320 | 8 | “ | “ | no | 28 | G | 2.04 | 13.5 | 30 | 0.3 | 1045 | 52.2 | 4.49 |
| 1 | 354 | 1 | Moose L. (CA) | “ | no | 28 | G | 1.94 | 5.6 | 19.8 | 40.8 | 755 | 54.28 | -110.87 |
| 1 | 394 | 8 | “ | “ | no | 28 | G | 2.15 | 5.6 | 19.8 | 40.8 | 755 | 54.28 | -110.87 |
| 1 | 759 | 40 | São Domingos (PT) | “ | no^1^ | 28 | G | 1.94 | 37.4 | 42.5 | 0.96 | 40 | 39.2 | -9.19 |
| 1 | 760 | 42 | “ | “ | no | 28 | G | 1.87 | 37.4 | 42.5 | 0.96 | 40 | 39.2 | -9.19 |
| 1 | 781 | 8 | L. Nero (RU) | “ | no | 28 | G | 1.81 | 1.6 | 4.7 | 58 | 1253 | 57.16 | 39.44 |
| 1 | 782 | 47 | São Domingos (PT) | “ | no | 28 | G | 1.51 | 37.4 | 42.5 | 0.96 | 40 | 39.2 | -9.19 |
| 1 | 787 | 40 | “ | “ | no^1^ | 28 | G | 1.62 | 37.4 | 42.5 | 0.96 | 40 | 39.2 | -9.19 |
| 1 | 788 | 61 | “ | “ | no | 28 | G | 2.15 | 37.4 | 42.5 | 0.96 | 40 | 39.2 | -9.19 |
| 1 | 790 | 8 | “ | “ | no | 28 | G | 2.12 | 37.4 | 42.5 | 0.96 | 40 | 39.2 | -9.19 |
| 1 | 826 | 40 | “ | “ | no^1^ | 28 | G | nd | 37.4 | 42.5 | 0.96 | 40 | 39.2 | -9.19 |
| 1 | 828 | 6 | L. Nero (RU) | “ | no | 28 | G | 1.96 | 1.6 | 4.7 | 58 | 1253 | 57.16 | 39.44 |
| 1 | 829 | 36 | “ | “ | no | 28 | G | 1.99 | 1.6 | 4.7 | 58 | 1253 | 57.16 | 39.44 |
| 1 | 836 | 8 | “ | “ | no | 28 | G | 1.67 | 1.6 | 4.7 | 58 | 1253 | 57.16 | 39.44 |
| 1 | 837 | 8 | “ | “ | no | 28 | G | nd | 1.6 | 4.7 | 58 | 1253 | 57.16 | 39.44 |
| 1 | 2A | 12 | L. Markusbölefjärden (FI) | “ | no | 28 | G | 2.21 | 4.5 | 9 | 1.56 | 13.8 | 60.24 | 19.93 |
| 1 | CCAP1459/15 | 8 | Lough Neagh (UK) | “ | no | 28;20 | G | 1.99 | 9 | 34 | 396 | 5700 | 54.60 | -6.42 |
| 1 | CYA126/8 | 22 | L. Langsjön (FI) | “ | yes | 28 | G | 1.99 | 9 | 18 | 1.43 | 16.3 | 60.24 | 19.95 |
| 1 | PCC7805 | 4 | Veluwemeer (NL) | “ | no | 28 | G | 2.26 | 1.5 | 5 | 32.8 | nd | 52.4 | 5.71 |
| 1 | PCC7811 | 8 | Paris, Vert le Petit (FR) | “ | no | 28 | G | 2.04 | 2 | 2 | 0.4 | nd | 48.55 | 2.37 |
| 1 | PH22 | 8 | L. Bagsværd Sø (DK) | “ | no | 28 | G | nd | 2 | 3 |  |  | 55.77 | 12.47 |
| 1 | SAG5.81 | 14 | Kiessee (DE) | “ | no | 28 | G | 2.18 | 1 | 2 | 0.15 | 0.12 | 51.52 | 9.92 |
| 1 | SAG6.89 | 11 | Plußsee (DE) | “ | yes | 28 | G | 2.01 | 9 | 30 | 0.14 | 0.46 | 54.18 | 10.44 |
| 1A | 277 | 50 | Wannsee (DE) | “ | no | 28 | G | 1.90 | 6 | 9 | 2.8 | 7000 | 52.43 | 13.2 |
| 1A | 364 | 3 | Moose L. (CA) | “ | no | 28 | G | 1.87 | 5.6 | 19.8 | 40.8 | 755 | 54.28 | -110.87 |
| 1A | 365 | 3 | “ | “ | no | 28 | G | 1.99 | 5.6 | 19.8 | 40.8 | 755 | 54.28 | -110.87 |
| 1A | 371 | 2 | “ | *rub.* | no | 28 | R | 0.77 | 5.6 | 19.8 | 40.8 | 755 | 54.28 | -110.87 |
| 1A | 372 | 2 | “ | “ | no | 28 | R | 0.65 | 5.6 | 19.8 | 40.8 | 755 | 54.28 | -110.87 |
| 1A | 384 | 19 | “ | *aga.* | no | 28 | G | 1.96 | 5.6 | 19.8 | 40.8 | 755 | 54.28 | -110.87 |
| 1A | 390 | 21 | “ | “ | no^1^ | 28 | G | 2.04 | 5.6 | 19.8 | 40.8 | 755 | 54.28 | -110.87 |
| 1A | 396 | 2 | “ | *rub.* | no | 28 | R | 0.45 | 5.6 | 19.8 | 40.8 | 755 | 54.28 | -110.87 |
| 1A | 552 | 2 | “ | “ | no^1^ | nd | R | 0.61 | 5.6 | 19.8 | 40.8 | 755 | 54.28 | -110.87 |
| 1A | 553 | 5 | “ | “ | no | 28 | R | 0.47 | 5.6 | 19.8 | 40.8 | 755 | 54.28 | -110.87 |
| 1A | 557 | 2 | “ | “ | no | 28 | R | 0.44 | 5.6 | 19.8 | 40.8 | 755 | 54.28 | -110.87 |
| 1B | 67 | 27 | Wörthersee (AT) | “ | Yes^3^ | 20 | R | 0.28 | 42 | 86 | 19.4 | 162 | 46.63 | 14.15 |
| 1B | 82 | 28 | Ammersee (DE) | “ | yes | 20 | R | 0.31 | 47 | 81 | 47 | 994 | 48.01 | 11.11 |
| 1B | 83/2 | 28 | “ | “ | yes | 20 | R | 0.26 | 47 | 81 | 47 | 994 | 48.01 | 11.11 |
| 2 | 3 | 10 | Mondsee (AT) | “ | yes | 20;16 | R | 0.26 | 37 | 68 | 14.2 | 247 | 47.84 | 13.37 |
| 2 | 40 | 17 | “ | “ | yes^3^ | 20;16 | R | 0.28 | 37 | 68 | 14.2 | 247 | 47.84 | 13.37 |
| 2 | 64 | 20 | Wörthersee (AT) | “ | yes | 20;16 | R | 0.28 | 42 | 86 | 19.4 | 162 | 46.63 | 14.15 |
| 2 | 80 | 17 | Schwarzensee (AT) | “ | yes | 20 | R | 0.42 | 27 | 54 | 0.5 | 9 | 47.75 | 13.5 |
| 2 | 97 | 17 | Mondsee (AT) | “ | yes | 20;16 | R | 0.24 | 37 | 68 | 14.2 | 247 | 47.84 | 13.37 |
| 2 | 108 | 23 | Irrsee (AT) | “ | yes | 20;16 | R | 0.17 | 15 | 32 | 3.6 | 28 | 47.91 | 13.31 |
| 2 | 110 | 44 | Mondsee (AT) | “ | yes^3^ | 20;16 | R | 0.28 | 37 | 68 | 14.2 | 247 | 47.84 | 13.37 |
| 2 | 111 | 10 | “ | “ | yes | 20;16 | R | 0.29 | 37 | 68 | 14.2 | 247 | 47.84 | 13.37 |
| 2 | 139 | 30 | Grabensee (AT) | “ | yes^3^ | 28;20 | R | 0.26 | 7 | 13 | 1.3 | 65 | 47.99 | 13.1 |
| 2 | 145 | 51 | “ | “ | yes^3^ | 20 | R | 0.23 | 7 | 13 | 1.3 | 65 | 47.99 | 13.1 |
| 2 | 161 | 30 | “ | “ | yes^3^ | 28;20 | R | 0.31 | 7 | 13 | 1.3 | 65 | 47.99 | 13.1 |
| 2 | 166 | 30 | “ | “ | yes^3^ | 28 | R | 0.24 | 7 | 13 | 1.3 | 65 | 47.99 | 13.1 |
| 2 | 169 | 30 | “ | “ | yes^3^ | 28;20 | R | 0.23 | 7 | 13 | 1.3 | 65 | 47.99 | 13.1 |
| 2 | 170 | 30 | “ | “ | yes^3^ | 20 | R | 0.24 | 7 | 13 | 1.3 | 65 | 47.99 | 13.1 |
| 2 | 178 | 30 | “ | “ | yes^3^ | 28 | R | 0.22 | 7 | 13 | 1.3 | 65 | 47.99 | 13.1 |
| 2 | 260 | 18 | Wannsee (DE) | *aga.* | yes | 28 | G | 2.11 | 6 | 9 | 2.8 | 7000 | 52.43 | 13.2 |
| 2 | 403 | 13 | Moore (Crane) L. (CA) | *rub.* | yes^3^ | 20 | R | 0.34 | 8.3 | 26 | 9.28 | 37.1 | 54.52 | -110.52 |
| 2 | 405 | 17 | “ | “ | yes^3^ | 20 | R | 0.30 | 8.3 | 26 | 9.28 | 37.1 | 54.52 | -110.52 |
| 2 | 406 | 13 | “ | “ | yes^3^ | 20 | R | 0.25 | 8.3 | 26 | 9.28 | 37.1 | 54.52 | -110.52 |
| 2 | 549 | 13 | “ | “ | yes^3^ | 20 | R | 0.29 | 8.3 | 26 | 9.28 | 37.1 | 54.52 | -110.52 |
| 2 | 550 | 13 | “ | “ | yes^3^ | 20 | R | 0.34 | 8.3 | 26 | 9.28 | 37.1 | 54.52 | -110.52 |
| 2 | 551 | 13 | “ | “ | yes^3^ | 20 | R | 0.28 | 8.3 | 26 | 9.28 | 37.1 | 54.52 | -110.52 |
| 2 | 761 | 33 | R. Garcia (IT) | “ | yes | 28 | R | 0.30 | 10 | 43 | 5.9 | 204 | 37.79 | 13.12 |
| 2 | 762 | 33 | “ | “ | yes | 28 | R | 0.33 | 10 | 43 | 5.9 | 204 | 37.79 | 13.12 |
| 2 | 775 | 32 | R. Nicolletti (IT) | “ | yes | 28 | R | 0.24 | 9 | 36 | 1.8 | 50 | 37.61 | 14.34 |
| 2 | 776 | 32 | “ | “ | yes | 28 | R | 0.25 | 9 | 36 | 1.8 | 50 | 37.61 | 14.34 |
| 2 | 777 | 32 | “ | “ | yes | 28 | R | 0.24 | 9 | 36 | 1.8 | 50 | 37.61 | 14.34 |
| 2 | 778 | 32 | “ | “ | yes | 28 | R | 0.24 | 9 | 36 | 1.8 | 50 | 37.61 | 14.34 |
| 2 | 779 | 53 | “ | “ | yes | 28 | R | 0.23 | 9 | 36 | 1.8 | 50 | 37.61 | 14.34 |
| 2 | 804 | 24 | Winnecook L. (US) | *aga.* | yes | 20 | G | 2.32 | 5.2 | 12.5 | 10 | 130 | 44.63 | -69.35 |
| 2 | 805 | 31 | “ | “ | yes | 20 | G | 1.69 | 5.2 | 12.5 | 10 | 130 | 44.63 | -69.35 |
| 2 | 806 | 41 | “ | “ | yes | 20 | G | 2.15 | 5.2 | 12.5 | 10 | 130 | 44.63 | -69.35 |
| 2 | 807 | 24 | “ | “ | yes | 20 | G | 1.13 | 5.2 | 12.5 | 10 | 130 | 44.63 | -69.35 |
| 2 | 808 | 25 | “ | “ | yes | 28;20 | G | 2.08 | 5.2 | 12.5 | 10 | 130 | 44.63 | -69.35 |
| 2 | 811 | 41 | “ | “ | yes | nd | G | 1.91 | 5.2 | 12.5 | 10 | 130 | 44.63 | -69.35 |
| 2 | 813 | 31 | China L. (US) | “ | yes | nd | G | nd | 8.53 | 25.9 | 16 | 67.6 | 44.44 | -69.58 |
| 2 | 814 | 43 | “ | “ | yes | 20 | G | 1.95 | 8.53 | 25.9 | 16 | 67.6 | 44.44 | -69.58 |
| 2 | 815 | 24 | “ | *rub.* | yes | 28;20;16 | R | 0.27 | 8.53 | 25.9 | 16 | 67.6 | 44.44 | -69.58 |
| 2 | 816 | 17 | “ | “ | yes | 28;20 | R | 0.26 | 8.53 | 25.9 | 16 | 67.6 | 44.44 | -69.58 |
| 2 | 830 | 32 | R. Garcia (IT) | “ | yes | 28 | R | 0.24 | 10 | 43 | 5.9 | 204 | 37.79 | 13.12 |
| 2 | 838 | 32 | “ | “ | yes | 28 | R | 0.19 | 10 | 43 | 5.9 | 204 | 37.79 | 13.12 |
| 2 | 839 | 32 | “ | “ | yes | 28 | R | 0.24 | 10 | 43 | 5.9 | 204 | 37.79 | 13.12 |
| 2 | 840 | 32 | “ | “ | yes | 28 | R | 0.24 | 10 | 43 | 5.9 | 204 | 37.79 | 13.12 |
| 2 | 21- | 33 | Figur (AT) | “ | yes | 28 | R | 0.26 | 8 | 12 | 0.08 | 0.1 | 48.11 | 16.32 |
| 2 | 496/1 | 13 | Moore (Crane) L. (CA) | “ | yes^3^ | 20 | R | 0.26 | 8.3 | 26 | 9.28 | 37.1 | 54.31 | -110.31 |
| 2 | 91/1 | 10 | Mondsee (AT) | “ | yes^3^ | 20;16 | R | 0.27 | 37 | 68 | 14.2 | 247 | 47.84 | 13.37 |
| 2 | A7 | 20 | Zürichsee (CH) | “ | yes | 20 | R | 0.25 | 52 | 136 | 65 | 1829 | 47.32 | 8.56 |
| 2 | CCAP1459/11A | 48 | L. Windermere (UK) | *aga.* | yes | 20 | G | 2.38 | 21 | 64 | 14.8 | 231 | 54.4 | -2.95 |
| 2 | CCAP1459/14 | 49 | Loughrigg Tarn (UK) | *rub.* | yes | 28;20 | R | 0.32 | 6.9 | 10.3 | 0.07 | 0.95 | 54.43 | -3.01 |
| 2 | CCAP1459/16 | 39 | Blelham Tarn (UK) | *aga.* | yes | 28;20 | G | 1.96 | 7 | 15 | 0.1 | 4.3 | 54.4 | -2.98 |
| 2 | CCAP1459/17 | 39 | “ | “ | yes | 28;20 | G | 1.92 | 7 | 15 | 0.1 | 4.3 | 54.4 | -2.98 |
| 2 | CCAP1459/21 | 52 | Esthwaite Water (UK) | “ | yes | 28;20 | G | 1.85 | 6.4 | 16 | 1.1 | 17.1 | 54.36 | -2.98 |
| 2 | CCAP1459/30 | 35 | Plöner See (DE) | *rub.* | yes | 20 | R | 0.26 | 16 | 60 | 30 | 393 | 54.12 | 10.41 |
| 2 | CCAP1459/31 | 17 | White Lough (IR) | *aga.* | yes | 28 | G | 1.82 | 6.15 | 10.7 | 0.074 | 1.076 | 54.11 | -6.96 |
| 2 | CCAP1460/5 | 39 | L. Kasumigaura (JP) | “ | yes | 28 | G | 1.90 | 4 | 10 | 220 | 1915 | 36.04 | 140.4 |
| 2 | PCC7821 | 26 | L. Gjersjoen (NO) | *rub.* | yes | 28 | R | 0.24 | 23 | 64 | 2.68 | 84.5 | 62.50 | 10.67 |
| 2A | 757 | 45 | L. Hormajärvi (FI) | *aga.* | yes | 28 | G | 2.25 | 7.3 | 21 | 5.1 | 16 | 60.29 | 24.01 |
| 2A | 758 | 45 | “ | “ | yes | 28 | G | 2.37 | 7.3 | 21 | 5.1 | 16 | 60.29 | 24.01 |
| 2A | 763 | 45 | “ | “ | yes | 28 | G | 2.44 | 7.3 | 21 | 5.1 | 16 | 60.29 | 24.01 |
| 2A | 764 | 45 | “ | “ | yes | 28 | G | 2.20 | 7.3 | 21 | 5.1 | 16 | 60.29 | 24.01 |
| 2A | 765 | 45 | “ | “ | yes | 28 | G | 2.34 | 7.3 | 21 | 5.1 | 16 | 60.29 | 24.01 |
| 2A | 766 | 58 | “ | “ | yes | 28 | G | 2.39 | 7.3 | 21 | 5.1 | 16 | 60.29 | 24.01 |
| 2A | 769 | 45 | “ | “ | yes | 28 | G | 2.39 | 7.3 | 21 | 5.1 | 16 | 60.29 | 24.01 |
| 2A | 770 | 45 | “ | “ | yes | 28 | G | 2.29 | 7.3 | 21 | 5.1 | 16 | 60.29 | 24.01 |
| 2A | 771 | 45 | “ | “ | yes | 28 | G | 2.33 | 7.3 | 21 | 5.1 | 16 | 60.29 | 24.01 |
| 2A | 772 | 45 | “ | “ | yes | 28 | G | 2.32 | 7.3 | 21 | 5.1 | 16 | 60.29 | 24.01 |
| 2A | 822 | 56 | L. Pyhäjärvi (FI) | “ | yes | nd | G | nd | 10 | 35 | 0.95 | nd | 61.45 | 23.47 |
| 2A | 863 | 55 | L. Hiidenvesi (FI) | “ | yes | nd | G | 2.10 | 6.6 | 33 | 30.3 | 935 | 60.38 | 24.19 |
| 2A | 865 | 55 | “ | “ | yes | 28 | G | 1.98 | 6.6 | 33 | 30.3 | 935 | 60.38 | 24.19 |
| 2A | 867 | 59 | “ | “ | yes | nd | G | 2.51 | 6.6 | 33 | 30.3 | 935 | 60.38 | 24.19 |
| 2A | 872 | 60 | “ | “ | yes | nd | G | 2.56 | 6.6 | 33 | 30.3 | 935 | 60.38 | 24.19 |
| 2A | 873 | 46 | “ | “ | yes | 28 | G | 2.25 | 6.6 | 33 | 30.3 | 935 | 60.38 | 24.19 |
| 2A | CCAP1459/36 | 57 | L. Gjersjoen (NO) | “ | yes^3^ | 28 | G | 1.98 | 23 | 64 | 2.68 | 84.5 | 62.50 | 10.67 |
| 3 | 704 | 54 | Saka (UG) | *pse.* | no^1^ | 28 | G | 2.23 | 3.6 | 8 | 1.4 | 10 | 0.69 | 30.24 |
| 3 | 707 | 54 | “ | “ | no^1^ | 28 | G | 2.25 | 3.6 | 8 | 1.4 | 10 | 0.69 | 30.24 |
| 3 | 708 | 54 | “ | “ | no^1^ | 28;20 | G | 2.26 | 3.6 | 8 | 1.4 | 10 | 0.69 | 30.24 |
| 3 | 710 | 54 | “ | “ | no^1^ | 28;20;16 | G | 2.47 | 3.6 | 8 | 1.4 | 10 | 0.69 | 30.24 |
| 3 | 711 | 54 | “ | “ | no^1^ | 28 | G | 2.21 | 3.6 | 8 | 1.4 | 10 | 0.69 | 30.24 |
| 3 | 712 | 54 | “ | “ | no^1^ | 28 | G | 2.26 | 3.6 | 8 | 1.4 | 10 | 0.69 | 30.24 |
| 3 | 713 | 54 | “ | “ | no^1^ | 28;20;16 | G | 2.04 | 3.6 | 8 | 1.4 | 10 | 0.69 | 30.24 |

^1^ Presence of the 5’ flanking region of the *mcy* gene cluster and smallest remnant of *mcy*T (Suppl. Figure 1)

^2^ Presence of the 5’ flanking region of the *mcy* gene cluster

^3^ Full *mcy* gene cluster present but MC synthesis is inactive (Christiansen *et al*., 2006)

^4^ G; green-pigmented; R; red-pigmented

**Suppl. Table 2.** List of 95 oligopeptides and frequency of occurrence in different lineages (peptides with single occurrence have been excluded). The grey shaded boxes indicate peptides that show the maximum occurrence among strains of a specific lineage (as depicted in Figure 4).

|  |  |  |  | | |  | Lineages | | |  |  | |  |  |  | |
| --- | --- | --- | --- | --- | --- | --- | --- | --- | --- | --- | --- | --- | --- | --- | --- | --- |
| Peptide No | Peptide Name | Ret time (min) | M+H | | | TOTAL | 1 | 1A | 2 | | | 2A | | 3 | 4 | |
| I. sulfated aeruginosins (MH - SO_3_) | | | |  | | | | | | | | | | | |  |
| .2 | uk | 7.65 | 821.10 | | | 5 | 0 | 5 | 0 | | | 0 | | 0 | 0 | |
| .3 | uk | 8.90 | 845.20 | | | 6 | 0 | 5 | 1 | | | 0 | | 0 | 0 | |
| .4 | uk | 9.69 | 689.20 | | | 3 | 2 | 1 | 0 | | | 0 | | 0 | 0 | |
| .5 | uk | 9.60 | 811.20 | | | 4 | 0 | 0 | 4 | | | 0 | | 0 | 0 | |
| .9 | uk | 11.59 | 771.20 | | | 14 | 7 | 0 | 7 | | | 0 | | 0 | 0 | |
| .12 | uk | 12.10 | 835.20 | | | 2 | 0 | 2 | 0 | | | 0 | | 0 | 0 | |
| .13 | uk | 12.54 | 795.10 | | | 15 | 0 | 0 | 15 | | | 0 | | 0 | 0 | |
| II. chlorinated/sulfated aeruginosins (Cl^-^/MH-SO_3_) | | | |  | | | | | | | | | | | |  |
| .18 | uk | 7.59 | 821.10 | | | 5 | 0 | 0 | 5 | | | 0 | | 0 | 0 | |
| .19 | Aeruginosin 89A/B^+^ | 7.71 | 717.20 | | | 2 | 2 | 0 | 0 | | | 0 | | 0 | 0 | |
| .21 | uk | 8.02 | 735.20 | | | 2 | 2 | 0 | 0 | | | 0 | | 0 | 0 | |
| .23 | uk | 8.86 | 845.00 | | | 5 | 0 | 0 | 5 | | | 0 | | 0 | 0 | |
| .24 | Aeruginosin 89A/B^+^ | 9.20 | 717.20 | | | 3 | 3 | 0 | 0 | | | 0 | | 0 | 0 | |
| .25 | uk | 10.38 | 733.20 | | | 4 | 4 | 0 | 0 | | | 0 | | 0 | 0 | |
| .26 | Aeruginosin 89A/B^+^ | 10.76 | 717.20 | | | 13 | 13 | 0 | 0 | | | 0 | | 0 | 0 | |
| .27 | uk | 10.82 | 715.20 | | | 8 | 8 | 0 | 0 | | | 0 | | 0 | 0 | |
| .29 | uk | 11.50 | 751.20 | | | 2 | 2 | 0 | 0 | | | 0 | | 0 | 0 | |
| .30 | uk | 11.70 | 879.20 | | | 2 | 0 | 2 | 0 | | | 0 | | 0 | 0 | |
| .32 | uk | 12.11 | 687.20 | | | 3 | 3 | 0 | 0 | | | 0 | | 0 | 0 | |
| .33 | Aeruginosin 98A | 12.10 | 689.20 | | | 2 | 2 | 0 | 0 | | | 0 | | 0 | 0 | |
| .34 | Aeruginosin 205A/B^+^ | 12.39 | 805.20 | | | 7 | 6 | 0 | 1 | | | 0 | | 0 | 0 | |
| .35 | uk | 12.60 | 829.00 | | | 3 | 1 | 0 | 2 | | | 0 | | 0 | 0 | |
| .36 | Aeruginosin 205A/B^+^ | 13.59 | 805.20 | | | 17 | 6 | 0 | 11 | | | 0 | | 0 | 0 | |
| .37 | uk | 14.50 | 835.20 | | | 2 | 0 | 2 | 0 | | | 0 | | 0 | 0 | |
| .38 | Aeruginosin A828* | 14.76 | 829.00 | | | 10 | 0 | 0 | 10 | | | 0 | | 0 | 0 | |
| .39 | uk | 15.20 | 869.10 | | | 3 | 1 | 2 | 0 | | | 0 | | 0 | 0 | |
| .41 | uk | 16.77 | 759.20 | | | 2 | 2 | 0 | 0 | | | 0 | | 0 | 0 | |
| .44 | uk | 17.60 | 893.00 | | | 2 | 0 | 2 | 0 | | | 0 | | 0 | 0 | |
| III. chlorinated aeruginosins (Cl^-^) | | | | |  | | | | | | | | | | |  |
| .47 | uk | 9.94 | 741.50 | | | 3 | 3 | 0 | 0 | | | 0 | | 0 | 0 | |
| .49 | uk | 11.52 | 775.40 | | | 4 | 4 | 0 | 0 | | | 0 | | 0 | 0 | |
| .51 | uk | 14.04 | 749.50 | | | 4 | 2 | 0 | 2 | | | 0 | | 0 | 0 | |
| .52 | uk | 14.74 | 607.20 | | | 3 | 3 | 0 | 0 | | | 0 | | 0 | 0 | |
| .53 | Aeruginosin KY608 | 15.00 | 609.20 | | | 2 | 2 | 0 | 0 | | | 0 | | 0 | 0 | |
| .55 | uk | 14.76 | 725.50 | | | 7 | 6 | 0 | 0 | | | 0 | | 0 | 1 | |
| .59 | Oscillaginin A | 20.99 | 615.20 | | | 6 | 0 | 6 | 0 | | | 0 | | 0 | 0 | |
| .60 | uk | 21.05 | 599.40 | | | 6 | 0 | 6 | 0 | | | 0 | | 0 | 0 | |
| .61 | uk | 21.32 | 629.40 | | | 6 | 0 | 6 | 0 | | | 0 | | 0 | 0 | |
| .63 | uk | 22.70 | 663.20 | | | 5 | 0 | 5 | 0 | | | 0 | | 0 | 0 | |
| .64 | uk | 23.11 | 613.40 | | | 6 | 0 | 6 | 0 | | | 0 | | 0 | 0 | |
| .65 | uk | 23.30 | 649.20 | | | 4 | 0 | 4 | 0 | | | 0 | | 0 | 0 | |
| .66 | uk | 23.91 | 627.20 | | | 4 | 0 | 4 | 0 | | | 0 | | 0 | 0 | |
| .68 | uk | 24.09 | 633.30 | | | 5 | 0 | 5 | 0 | | | 0 | | 0 | 0 | |
| .69 | uk | 25.20 | 647.40 | | | 6 | 0 | 6 | 0 | | | 0 | | 0 | 0 | |
| .70 | uk | 26.20 | 661.30 | | | 2 | 0 | 2 | 0 | | | 0 | | 0 | 0 | |
| IV. Microcystins | | | | |  | | | | | | | | | | |  |
| .71 | unknown MC | 19.57 | 886.6 | | | 2 | 2 | 0 | 0 | | | 0 | | 0 | 0 | |
| .72 | D-Me-MC-RR | 21.24 | 1024.60 | | | 54 | 8 | 0 | 28 | | | 16 | | 0 | 2 | |
| .73 | D-Me-MC-HtyR | 26.28 | 1045.70 | | | 43 | 6 | 0 | 24 | | | 11 | | 0 | 2 | |
| .74 | D-Me-MC-LR | 27.09 | 981.60 | | | 55 | 8 | 0 | 29 | | | 16 | | 0 | 2 | |
| .75 | D-Me-MC-YR | 29.74 | 1031.70 | | | 11 | 0 | 0 | 1 | | | 10 | | 0 | 0 | |
| V. Anabaenopeptins | | | | |  | | | | | | | | | | |  |
| .76 | Anabaenopeptin C | 16.05 | 809.50 | | | 34 | 3 | 8 | 8 | | | 15 | | 0 | 0 | |
| .77 | D-Me-Anabaenopeptin B | 16.52 | 823.60 | | | 28 | 4 | 7 | 2 | | | 15 | | 0 | 0 | |
| .78 | Anabaenopeptin B | 16.74 | 837.60 | | | 96 | 14 | 10 | 52 | | | 17 | | 0 | 3 | |
| .79 | Anabaenopeptin E/F^+^ | 18.51 | 851.60 | | | 68 | 9 | 7 | 32 | | | 17 | | 0 | 3 | |
| .80 | Anabaenopeptin A | 22.65 | 844.40 | | | 60 | 5 | 9 | 43 | | | 1 | | 0 | 2 | |
| .81 | Oscillamide Y | 24.51 | 858.50 | | | 41 | 4 | 8 | 26 | | | 1 | | 0 | 2 | |
| VI. sulfated cyanopeptolins (MH-SO_3_ - H_2_O) | | | | |  | | | | | | | | | | |  |
| .82 | uk | 11.65 | 1079.20 | | | 4 | 0 | 0 | 1 | | | 0 | | 0 | 3 | |
| .83 | Oscillapeptin J | 14.14 | 1093.10 | | | 3 | 0 | 0 | 1 | | | 0 | | 0 | 2 | |
| .85 | uk | 15.80 | 1109.10 | | | 4 | 0 | 0 | 0 | | | 4 | | 0 | 0 | |
| .86 | uk | 16.57 | 1063.10 | | | 2 | 0 | 0 | 2 | | | 0 | | 0 | 0 | |
| .88 | uk | 18.29 | 1077.20 | | | 2 | 0 | 0 | 2 | | | 0 | | 0 | 0 | |
| .95 | Cyanopeptolin 975 | 20.45 | 1091.20 | | | 3 | 0 | 0 | 3 | | | 0 | | 0 | 0 | |
| .98 | uk | 22.58 | 977.10 | | | 3 | 3 | 0 | 0 | | | 0 | | 0 | 0 | |
| .101 | uk | 22.80 | 1093.00 | | | 5 | 0 | 0 | 0 | | | 5 | | 0 | 0 | |
| .102 | uk | 23.90 | 1061.00 | | | 2 | 0 | 0 | 2 | | | 0 | | 0 | 0 | |
| .103 | uk | 24.12 | 1154.00 | | | 5 | 5 | 0 | 0 | | | 0 | | 0 | 0 | |
| .104 | uk | 24.20 | 1075.00 | | | 4 | 2 | 0 | 2 | | | 0 | | 0 | 0 | |
| .108 | Oscillapeptin F | 26.00 | 1089.10 | | | 14 | 9 | 5 | 0 | | | 0 | | 0 | 0 | |
| .109 | uk | 26.00 | 1163.00 | | | 2 | 2 | 0 | 0 | | | 0 | | 0 | 0 | |
| .110 | Oscillapeptin D | 26.10 | 1128.00 | | | 3 | 3 | 0 | 0 | | | 0 | | 0 | 0 | |
| .111 | Lyngbyastatin 4 | 26.30 | 1137.20 | | | 2 | 0 | 2 | 0 | | | 0 | | 0 | 0 | |
| .112 | uk | 26.43 | 1103.20 | | | 7 | 7 | 0 | 0 | | | 0 | | 0 | 0 | |
| .113 | uk | 27.68 | 977.00 | | | 2 | 2 | 0 | 0 | | | 0 | | 0 | 0 | |
| .115 | uk | 28.48 | 1171.10 | | | 2 | 0 | 2 | 0 | | | 0 | | 0 | 0 | |
| .118 | uk | 29.84 | 981.00 | | | 9 | 0 | 0 | 9 | | | 0 | | 0 | 0 | |
| .119 | Cyanopeptolin 960 | 29.97 | 961.10 | | | 4 | 3 | 0 | 1 | | | 0 | | 0 | 0 | |
| .122 | Oscillapeptin E/CP1138^+^ | 31.58 | 1138.00 | | | 6 | 6 | 0 | 0 | | | 0 | | 0 | 0 | |
| VII. cyanopeptolins (MH - H_2_O) | | | | |  | | | | | | | | | | |  |
| .126 | uk | 17.72 | 1034.20 | | | 7 | 0 | 0 | 7 | | | 0 | | 0 | 0 | |
| .127 | uk | 14.20 | 999.20 | | | 3 | 0 | 0 | 3 | | | 0 | | 0 | 0 | |
| .128 | uk | 15.55 | 861.40 | | | 2 | 0 | 0 | 2 | | | 0 | | 0 | 0 | |
| .129 | uk | 15.88 | 831.50 | | | 2 | 0 | 0 | 2 | | | 0 | | 0 | 0 | |
| .130 | Micropeptin SD 944 | 14.42 | 945.30 | | | 10 | 0 | 0 | 0 | | | 10 | | 0 | 0 | |
| .131 | uk | 15.90 | 831.30 | | | 2 | 0 | 0 | 2 | | | 0 | | 0 | 0 | |
| .132 | Oscillapeptin G | 17.30 | 1112.30 | | | 2 | 0 | 0 | 2 | | | 0 | | 0 | 0 | |
| .133 | Micropeptin MZ 939A/B^+^ | 17.60 | 940.40 | | | 2 | 0 | 0 | 2 | | | 0 | | 0 | 0 | |
| .134 | uk | 18.07 | 863.30 | | | 7 | 0 | 0 | 7 | | | 0 | | 0 | 0 | |
| .135 | uk | 18.47 | 1012.30 | | | 2 | 1 | 1 | 0 | | | 0 | | 0 | 0 | |
| .136 | uk | 18.90 | 1105.60 | | | 2 | 0 | 0 | 2 | | | 0 | | 0 | 0 | |
| .137 | uk | 19.06 | 1084.40 | | | 10 | 0 | 0 | 0 | | | 10 | | 0 | 0 | |
| .140 | uk | 19.73 | 1048.60 | | | 7 | 0 | 0 | 7 | | | 0 | | 0 | 0 | |
| .143 | uk | 20.40 | 983.00 | | | 3 | 0 | 0 | 3 | | | 0 | | 0 | 0 | |
| .144 | Planktopeptin BL1125 | 20.62 | 1126.30 | | | 2 | 0 | 1 | 1 | | | 0 | | 0 | 0 | |
| .145 | uk | 20.83 | 1193.40 | | | 2 | 0 | 0 | 2 | | | 0 | | 0 | 0 | |
| .147 | Micropeptin EI 964  Micropeptin EI 964 | 23.4 | 965.10 | | | 5 | 0 | 0 | 5 | | | 0 | | 0 | 0 | |
| .150 | uk | 24.90 | 1053.20 | | | 5 | 0 | 0 | 5 | | | 0 | | 0 | 0 | |
| VIII. Planktocyclins | | | |  | | | | | | | | | | | |  |
| .158 | Planktocyclin sulfoxide | 13.90 | 817.40 | | | 10 | 2 | 0 | 8 | | | 0 | | 0 | 0 | |
| .159 | Planktocyclin | 32.36 | 801.50 | | | 23 | 5 | 0 | 17 | | | 0 | | 0 | 1 | |

* Kohler *et al*. (in press) ^+^ possible structural variants with identical masses; uk, unknown.

1 130

354 C*TGAGGTTGA* *TCAGGGGTTA* *TGAGTGATGG* *AAGACACAGG* *AATTGGGTGA* *TTTGTCCCAT* *TTTAATG*--- ---------- ---------- ---------- ---------- ---------- ----------

390 *GTGAGGTTGA* *TCAGGGGTTA* *TGAGTGATGG* *AAGACACAGG* *AATTGGGTGA* *TTTGTCCCAT* *TTTAATG*--- ---------- ---------- ---------- ---------- ---------- ----------

704 *GTGAGGTTGA* *TCAGGGGTTA* *TGAGTGATGG* *AAGACACAGG* *AATTGGGTGA* *TTTGTCCCAT* *TTTAATG*--- ---------- ---------- ---------- ---------- ---------- ----------

826 *GTGAGGTTGA* *TCAGGGGTTA* *TGAGTGATGG* *AAGACACAGG* *AATTGGGTGA* *TTTGTCCCAT* *TTTAATG*--- ---------- ---------- ---------- ---------- ---------- ----------

787 *GTGAGGTTGA* *TCAGGGGTTA* *TGAGTGATGG* *AAGACACAGG* *AATTGGGTGA* *TTTGTCCCAT* *TTTAATG*--- ---------- ---------- ---------- ---------- ---------- ----------

708 *GTGAGGTTGA* *TCAGGGGTTA* *TGAGTGATGG* *AAGACACAGG* *AATTGGGTGA* *TTTGTCCCAT* *TTTAATG*--- ---------- ---------- ---------- ---------- ---------- ----------

759 *GTGAGGTTGA* *TCAGGGGTTA* *TGAGTGATGG* *AAGACACAGG* *AATTGGGTGA* *TTTGTCCCAT* *TTTAATG*--- ---------- ---------- ---------- ---------- ---------- ----------

710 *GTGAGGTTGA* *TCAGGGGTTA* *TGAGTGATGG* *AAGACACAGG* *AATTGGGTGA* *TTTGTCCCAT* *TTTAATG*--- ---------- ---------- ---------- ---------- ---------- ----------

712 *GTGAGGTTGA* *TCAGGGGTTA* *TGAGTGATGG* *AAGACACAGG* *AATTGGGTGA* *TTTGTCCCAT* *TTTAATG*--- ---------- ---------- ---------- ---------- ---------- ----------

711 *GTGAGGTTGA* *TCAGGGGTTA* *TGAGTGATGG* *AAGACACAGG* *AATTGGGTGA* *TTTGTCCCAT* *TTTAATG*--- ---------- ---------- ---------- ---------- ---------- ----------

782 *GTGAGGTTGA* *TCAGGGGTTA* *TGAGTGATGG* *AAGACACAGG* *AATTGGGTGA* A*TTGTCCCAT* *TTTAATG*--- ---------- ---------- ---------- ---------- ---------- ----------

707 *GTGAGGTTGA* *TCAGGGGTTA* *TGAGTGATGG* *AAGACACAGG* *AATTGGGTGA* *TTTGTCCCAT* *TTTAATG*--- ---------- ---------- ---------- ---------- ---------- ----------

252 *GTGAGGTTGA* *TCAGGGGTTA* *TGAGTGATGG* *AAGACACAGG* *AATTGGGTGA* *TTTGTCCCAT* *TTTAATG*--- ---------- ---------- ---------- ---------- ---------- ----------

CYA126/8 *GTGAGGTTGA* *TCAGGGGTTA* *TGAGTGATGG* *AAGACACAGG* *AATTGGGTGA* *TTTGTCCCAT* *TTTAATG*--- ---------- ---------- ---------- ---------- ---------- ----------

713 *GTGAGGTTGA* *TCAGGGGTTA* *TG*G*GTGATGG* *AAGACACAGG* *AATTGGGTGA* *TTTGTCCCAT* *TTTAATG*TGA ATATTTGTAT TAGGGTGGGG AATGCGATCG CTCAATTCTA CGGAGATTTA TAGATAATGC

CYA98 *GTGAGGTTGA* *TCAGGGGTTA* *TG*G*GTGATGG* *AAGACACAGG* *AATTGGGTGA* *TTTGTCCCAT* *TTTAATG*TGA ATATTTGTAT TAGGGTGGGG AATGCGATCG CTCAATTCTA CGGAGATTTA TAGATAATGC

131 260

354 ---------- ---------- ---------- ---------- ---------- ---------- ---------- ---------- ---------- ---------- ---------- ---------- ----------

390 ---------- ---------- ---------- ---------- ---------- ---------- ---------- ---------- ---------- ---------- ---------- ---------- ----------

704 ---------- ---------- ---------- ---------- ---------- ---------- ---------- ---------- ---------- ---------- ---------- ---------- ----------

826 ---------- ---------- ---------- ---------- ---------- ---------- ---------- ---------- ---------- ---------- ---------- ---------- ----------

787 ---------- ---------- ---------- ---------- ---------- ---------- ---------- ---------- ---------- ---------- ---------- ---------- ----------

708 ---------- ---------- ---------- ---------- ---------- ---------- ---------- ---------- ---------- ---------- ---------- ---------- ----------

759 ---------- ---------- ---------- ---------- ---------- ---------- ---------- ---------- ---------- ---------- ---------- ---------- ----------

710 ---------- ---------- ---------- ---------- ---------- ---------- ---------- ---------- ---------- ---------- ---------- ---------- ----------

712 ---------- ---------- ---------- ---------- ---------- ---------- ---------- ---------- ---------- ---------- ---------- ---------- ----------

711 ---------- ---------- ---------- ---------- ---------- ---------- ---------- ---------- ---------- ---------- ---------- ---------- ----------

782 ---------- ---------- ---------- ---------- ---------- ---------- ---------- ---------- ---------- ---------- ---------- ---------- ----------

707 ---------- ---------- ---------- ---------- ---------- ---------- ---------- ---------- ---------- ---------- ---------- ---------- ----------

252 ---------- ---------- ---------- ---------- ---------- ---------- ---------- ---------- ---------- ---------- ---------- ---------- ----------

CYA126/8 ---------- ---------- ---------- ---------- ---------- ---------- ---------- ---------- ---------- ---------- ---------- ---------- ----------

713 AAACTGAAAC TAAAGTCTCT TTACGATCAC AAGAAAAAAC TAAAATATTC ATGGTTAATC CTCGTCCCAA AACTGCCAAA AAAACCAGAC AAATTCCCCT GAGATTACTA TTGATTGTCC CCTTTGTATT

CYA98 AAACTGAAAC TAAAGTCTCT TTACGATCAC AAGAAAAAAC TAAAATATTC ATGGTTAATC CTCGTCCCAA AACTGCCAAA AAAACCAGAC AAATTCCCCT GAGATTACTA TTGATTGTCC CCTTTGTATT

261 390

354 ---------- *GGCGCGGTAG* *GATTAGTTGG* *TTATCTTTCC* *TATCGTAGTG* *GACAAAAAGC* *GGTAGAAGAT* *ATGGCAAAAC* *CTTTGATGGT* *AGAAATCGGC* *GATCCCGATG* *TCCAAGTTGT* *AATAGAATTA*

390 ---------- *GGCGCGGTAG* *GATTAGTTGG* *TTATCTTTCC* *TATCGTAGTG* *GACAAAAAGC* *GGTAGAAGAT* *ATGGCAAAAC* *CTTTGATGGT* *AGAAATCGGC* *GATCCCGATG* *TCCAAGTTGT* *AATAGAATTA*

704 ---------- *GGCGCGGTAG* *GATTAGTTGG* *TTATCTTTCC* *TATCGTAGTG* *GACAAAAAGC* *GGTAGAAGAT* *ATGGCAAAAC* *CTTTGATGGT* *AGAAATCGGC* *GATCCCGATG* *TCCAAGTTGT* *AATAGAATTA*

826 ---------- *GGCGCGGTAG* *GATTAGTTGG* *TTATCTTTCC* *TATCGTAGTG* *GACAAAAAGC* *GGTAGAAGAT* *ATGGCAAAAC* *CTTTGATGGT* *AGAAATCGGC* *GATCCCGATG* *TCCAAGTTGT* *AATAGAATTA*

787 ---------- *GGCGCGGTAG* *GATTAGTTGG* *TTATCTTTCC* *TATCGTAGTG* *GACAAAAAGC* *GGTAGAAGAT* *ATGGCAAAAC* *CTTTGATGGT* *AGAAATCGGC* *GATCCCGATG* *TCCAAGTTGT* *AATAGAATTA*

708 ---------- *GGCGCGGTAG* *GATTAGTTGG* *TTATCTTTCC* *TATCGTAGTG* *GACAAAAAGC* *GGTAGAAGAT* *ATGGCAAAAC* *CTTTGATGGT* *AGAAATCGGC* *GATCCCGATG* *TCCAAGTTGT* *AATAGAATTA*

759 ---------- *GGCGCGGTAG* *GATTAGTTGG* *TTATCTTTCC* *TATCGTAGTG* *GACAAAAAGC* *GGTAGAAGAT* *ATGGCAAAAC* *CTTTGATGGT* *AGAAATCGGC* *GATCCCGATG* *TCCAAGTTGT* *AATAGAATTA*

710 ---------- *GGCGCGGTAG* *GATTAGTTGG* *TTATCTTTCC* *TATCGTAGTG* *GACAAAAAGC* *GGTAGAAGAT* *ATGGCAAAAC* *CTTTGATGGT* *AGAAATCGGC* *GATCCCGATG* *TCCAAGTTGT* *AATAGAATTA*

712 ---------- *GGCGCGGTAG* *GATTAGTTGG* *TTATCTTTCC* *TATCGTAGTG* *GACAAAAAGC* *GGTAGAAGAT* *ATGGCAAAAC* *CTTTGATGGT* *AGAAATCGGC* *GATCCCGATG* *TCCAAGTTGT* *AATAGAATTA*

711 ---------- *GGCGCGGTAG* *GATTAGTTGG* *TTATCTTTCC* *TATCGTAGTG* *GACAAAAAGC* *GGTAGAAGAT* *ATGGCAAAAC* *CTTTGATGGT* *AGAAATCGGC* *GATCCCGATG* *TCCAAGTTGT* *AATAGAATTA*

782 ---------- *GGCGCGGTAG* *GATTAGTTGG* *TTATCTTTCC* *TATCGTAGTG* *GACAAAAAGC* *GGTAGAAGAT* *ATGGCAAAAC* *CTTTGATGGT* *AGAAATCGGC* *GATCCCGATG* *TCCAAGTTGT* *AATAGAATTA*

707 ---------- *GGCGCGGTAG* *GATTAGTTGG* *TTATCTTTCC* *TATCGTAGTG* *GACAAAAAGC* *GGTAGAAGAT* *ATGGCAAAAC* *CTTTGATGGT* *AGAAATCGGC* *GATCCCGATG* *TCCAAGTTGT* *AATAGAATTA*

252 ---------- *GGCGCGGTAG* *GATTAGTTGG* *TTATCTTTCC* *TATCGTAGTG* *GACAAAAAGC* *GGTAGAAGAT* *ATGGCAAAAC* *CTTTGATGGT* *AGAAATCGGC* *GATCCCGATG* *TCCAAGTTGT* *AATAGAATTA*

CYA126/8 ---------- *GGCGCGGTAG* *GATTAGTTGG* *TTATCTTTCC* *TATCGTAGTG* *GACAAAAAGC* *GGTAGAAGAT* *ATGGCAAAAC* *CTTTGATGGT* *AGAAATCGGC* *GATCCCGATG* *TCCAAGTTGT* *AATAGAATTA*

713 GCAAATTGTA *GGCGCGGTAG* *GATTAGT*C*GG* *TTATCTTTCC* *TATCGTAGTG* *GACAAAAAGC* *GGTAGAAGAT* *ATGGCAAAAC* *CTTTGATGGT* *AGAAATCGGC* *GATCCCGATG* *TCCAAGTTGT* *AATAGAATTA*

CYA98 GCAAATTGTA *GGCGCGGTAG* *GATTAGT*C*GG* *TTATCTTTCC* *TATCGTAGTG* *GACAAAAAGC* *GGTAGAAGAT* *ATGGCAAAAC* *CTTTGATGGT* *AGAAATCGGC* *GATCCCGATG* *TCCAAGTTGT* *AATAGAATTA*

391 520

354 *ATTAAAGAAA* *TTCCAGATCC* *AGAAATTCCT* *CTAATCCAAT* *TTCTAACAAA* *AGCAGTGCGG* *AAATTTCAAT* *TTGAACAGAT* *AGTTGACTTA* *ATTGATCCCC* *TGATCAATGA* *TCAATAGCGA* *TTTTCCCAAG*

390 *ATTAAAGAAA* *TTCCAGATCC* *AGAAATTCCT* *CTAATCCAAT* *TTCTAACAAA* *AGCAGTGCGG* *AAATTTCAAT* *TTGAACAGAT* *AGTTGACTTA* *ATTGATCCCC* *TGATCAATGA* *TCAATAGCGA* *TTTTCCCAAG*

704 *ATTAAAGAAA* *TTCCAGATCC* *AGAAATTCCT* *CTAATCCAAT* *TTCTAACAAA* *AGCAGTGCGG* *AAATTTCAAT* *TTGAACAGAT* *AGTTGACTTA* *ATTGATCCCC* *TGATCAATGA* *TCAATAGCGA* *TTTTCCCAAG*

826 *ATTAAAGAAA* *TTCCAGATCC* *AGAAATTCCT* *CTAATCCAAT* *TTCTAACAAA* *AGCAGTGCGG* *AAATTTCAAT* *TTGAACAGAT* *AGTTGACTTA* *ATTGATCCCC* *TGATCAATGA* *TCAATAGCGA* *TTTTCCCAAG*

787 *ATTAAAGAAA* *TTCCAGATCC* *AGAAATTCCT* *CTAATCCAAT* *TTCTAACAAA* *AGCAGTGCGG* *AAATTTCAAT* *TTGAACAGAT* *AGTTGACTTA* *ATTGATCCCC* *TGATCAATGA* *TCAATAGCGA* *TTTTCCCAAG*

708 *ATTAAAGAAA* *TTCCAGATCC* *AGAAATTCCT* *CTAATCCAAT* *TTCTAACAAA* *AGCAGTGCGG* *AAATTTCAAT* *TTGAACAGAT* *AGTTGACTTA* *ATTGATCCCC* *TGATCAATGA* *TCAATAGCGA* *TTTTCCCAAG*

759 *ATTAAAGAAA* *TTCCAGATCC* *AGAAATTCCT* *CTAATCCAAT* *TTCTAACAAA* *AGCAGTGCGG* *AAATTTCAAT* *TTGAACAGAT* *AGTTGACTTA* *ATTGATCCCC* *TGATCAATGA* *TCAATAGCGA* *TTTTCCCAAG*

710 *ATTAAAGAAA* *TTCCAGATCC* *AGAAATTCCT* *CTAATCCAAT* *TTCTAACAAA* *AGCAGTGCGG* *AAATTTCAAT* *TTGAACAGAT* *AGTTGACTTA* *ATTGATCCCC* *TGATCAATGA* *TCAATAGCGA* *TTTTCCCAAG*

712 *ATTAAAGAAA* *TTCCAGATCC* *AGAAATTCCT* *CTAATCCAAT* *TTCTAACAAA* *AGCAGTGCGG* *AAATTTCAAT* *TTGAACAGAT* *AGTTGACTTA* *ATTGATCCCC* *TGATCAATGA* *TCAATAGCGA* *TTTTCCCAAG*

711 *ATTAAAGAAA* *TTCCAGATCC* *AGAAATTCCT* *CTAATCCAAT* *TTCTAACAAA* *AGCAGTGCGG* *AAATTTCAAT* *TTGAACAGAT* *AGTTGACTTA* *ATTGATCCCC* *TGATCAATGA* *TCAATAGCGA* *TTTTCCCAAG*

782 *ATTAAAGAAA* *TTCCAGATCC* *AGAAATTCCT* *CTAATCCAAT* *TTCTAACAAA* *AGCAGTGCGG* *AAATTTCAAT* *TTGAACAGAT* *AGTTGACTTA* *ATTGATCCCC* *TGATCAATGA* *TCAATAGCGA* *TTTTCCCAAG*

707 *ATTAAAGAAA* *TTCCAGATCC* *AGAAATTCCT* *CTAATCCAAT* *TTCTAACAAA* *AGCAGTGCGG* *AAATTTCAAT* *TTGAACAGAT* *A*A*TTGACTTA* *ATTGATCCCC* *TGATCAATGA* *TCAATAGCGA* *TTTTCCCAAG*

252 *ATTAAA*---- ---------- ---------- ---------- ---------- ---------- ---------- ---------- ---------- ---------- ---------- ---------- ----------

CYA126/8 *ATTAAAGAAA* *TTCCAGATCC* *AGAAATTCCT* *CTAATCCAAT* *TTCTAACAAA* *AGCAGTGCGG* *AAATTTCAAT* *TTGAACAGAT* *A*A*TTGACTTA* *ATTGATCCCC* *TGATCAATGA* *TCAATAGCGA* *TTTTCCCAAG*

713 *ATTAAAGAAA* *TTCCAGATCC* *AGAAATTCCT* *CTAATCCAAT* *TTCTAACAAA* *AGCAGTGCGG* *AAATTTCAAT* *TTGAACAGAT* *A*A*TTGACTTA* *ATTGATCCCC* *TGATCAATGA* *TCAATAGCGA* *TTTTCCCAAG*

CYA98 *ATTAAAGAAA* *TTCCAGATCC* *AGAAATTCCT* *CTAATCCAAT* *TTCTAACAAA* *AGCAGTGCGG* *AAATTTCAAT* *TTGAACAGAT* *A*A*TTGACTTA* *ATTGATCCCC* *TGATCAATGA* *TCAATAGCGA* *TTTTCCCAAG*

521 646

354 *CATTCTAGGA* *CAAGAGATTT* *GGCTGAGTGC* *AAAAAGAAAT* *GATCTCCGGG* *AAA*T*ATCTCC* *ACAGAGAAAG* *CCGAGTTGGT* *T*C*GTTCCCAC* *CAAGCTTCTA* *TCTCCAAGGC* *GTTAGGTTTC* *AAATCT*

390 *CATTCTAGGA* *CAAGAGATTT* *GGCTGAGTGC* *AAAAAGAAAT* *GATCTCCGGG* *AAACATCTCC* *ACAGAGAAAG* *CCGAGTTGGT* *TTGTTCCCAC* *CAAGCTTCTA* *TCTCCAAGGC* *GTTAGGTTTC* *AAATCT*

704 *CATTCTAGGA* *CAAGAGATTT* *GGCTGAGTGC* *AAAAAGAAAT* *GATCTCCGGG* *AAACATCTCC* *ACAGAGAAAG* *CCGAGTTGGT* *TTGTTCCCAC* *CAAGCTTCTA* *TCTCCAAGGC* *GTTAGGTTTC* *AAATCT*

826 *CATTCTAGGA* *CAAGAGATTT* *GGCTGAGTGC* *AAAAAGAAAT* *GATCTCCGGG* *AAACATCTCC* *ACAGAGAAAG* *CCGAGTTGGT* *TTGTTCCCAC* *CAAGCTTCTA* *TCTCCAAGGC* *GTTAGGTTTC* *AAATCT*

787 *CATTCTAGGA* *CAAGAGATTT* *GGCTGAGTGC* *AAAAAGAAAT* *GATCTCCGGG* *AAACATCTCC* *ACAGAGAAAG* *CCGAGTTGGT* *TTGTTCCCAC* *CAAGCTTCTA* *TCTCCAAGGC* *GTTAGGTTTC* *AAATCT*

708 *CATTCTAGGA* *CAAGAGATTT* *GGCTGAGTGC* *AAAAAGAAAT* *GATCTCCGGG* *AAACATCTCC* *ACAGAGAAAG* *CCGAGTTGGT* *TTGTTCCCAC* *CAAGCTTCTA* *TCTCCAAGGC* *GTTAGGTTTC* *AAATCT*

759 *CATTCTAGGA* *CAAGAGATTT* *GGCTGAGTGC* *AAAAAGAAAT* *GATCTCCGGG* *AAACATCTCC* *ACAGAGAAAG* *CCGAGTTGGT* *TTGTTCCCAC* *CAAGCTTCTA* *TCTCCAAGGC* *GTTAGGTTTC* *AAATCT*

710 *CATTCTAGGA* *CAAGAGATTT* *GGCTGAGTGC* *AAAAAGAAAT* *GATCTCCGGG* *AAACATCTCC* *ACAGAGAAAG* *CCGAGTTGGT* *TTGTTCCCAC* *CAAGCTTCTA* *TCTCCAAGGC* *GTTAGGTTTC* *AAATCT*

712 *CATTCTAGGA* *CAAGAGATTT* *GGCTGAGTGC* *AAAAAGAAAT* *GATCTCCGGG* *AAACATCTCC* *ACAGAGAAAG* *CCGAGTTGGT* *TTGTTCCCAC* *CAAGCTTCTA* *TCTCCAAGGC* *GTTAGGTTTC* *AAATCT*

711 *CATTCTAGGA* *CAAGAGATTT* *GGCTGAGTGC* *AAAAAGAAAT* *GATCTCCGGG* *AAACATCTCC* *ACAGAGAAAG* *CCGAGTTGGT* *TTGTTCCCAC* *CAAGCTTCTA* *TCTCCAAGGC* *GTTAGGTTTC* *AAATCT*

782 *CATTCTAGGA* *CAAGAGATTT* *GGCTGAGTGC* *AAAAAGAAAT* *GATCTCCGGG* *AAACATCTCC* *ACAGAGAAAG* *CCGAGTTGGT* *TTGTTCCCAC* *CAAGCTTCTA* *TCTCCAAGGC* *GTTAGGTTTC* *AAATCT*

707 *CATTCTAGGA* *CAAGAGATTT* *GGCTGAGTGC* *AAAAAGAAAT* *GATCTCCGGG* *AAACATCTCC* *ACAGAGAAAG* *CCGAGTTGGT* *TTGTTCCC*G*C* *CAAGCTTCTA* *TCTCCAAGGC* *GTTAGGTTTC* *AAATCT*

252 ---------- ---------- ---------- ---------- ---------- ---------- ---------- ---------- ---------- ---------- ---------- ---------- ------

CYA126/8 *CATTCTAGGA* *CAAGAGATTT* *GGCTGAGTGC* *AAAAAGAAAT* *GATCTCCGGG* *AAACATCTCC* *ACAGAGAAAG* *CCGAGTTGGT* *TTGTTCCC*G*C* *CAAGCTTCTA* *TCTCCAAGGC* *GTTAGGTTTC* *AAATCT*

713 *CATTCTAGGA* *CAAGAGATTT* *GGCTGAGTGC* *AAAAAGAAAT* *GATCTCCGGG* *AAACATCTCC* *ACAGAGAAAG* *CCGAGTTGGT* *TTGTTCCC*G*C* *CAAGCTTCTA* *TCTCCAAGGC* *GTTAGGTTTC* *AAATCT*

CYA98 *CATTCTAGGA* *CAAGAGATTT* *GGCTGAGTGC* *AAAAAGAAAT* *GATCTCCGGG* *AAACATCTCC* *ACAGAGAAAG* *CCGAGTTGGT* *TTGTTCCC*G*C* *CAAGCTTCTA* *TCTCCAAGGC* *GTTAGGTTTC* *AAATCT*

**Suppl. Figure 1.** Sequence alignment showing the 5´ end flanking regions of *mcy*T found in 15 nontoxic strains that lost the *mcy* gene cluster and the major part of *mcy*T. The remnant of the smallest part of *mcy*T is underlined (Pos. 449-615 of the *mcy* gene cluster of the toxic strain CYA126/8; AJ441056). One strain No713 contained an insertion (202 bp) which is identical to the 5’ flanking region of the *mcy* gene cluster of *P. rubescens* CYA98 (AM990462; Rounge *et al*., 2009). Strain No252 lost the *mcy*T gene completely (deletion type IV as described in Christiansen *et al*., 2008).

**
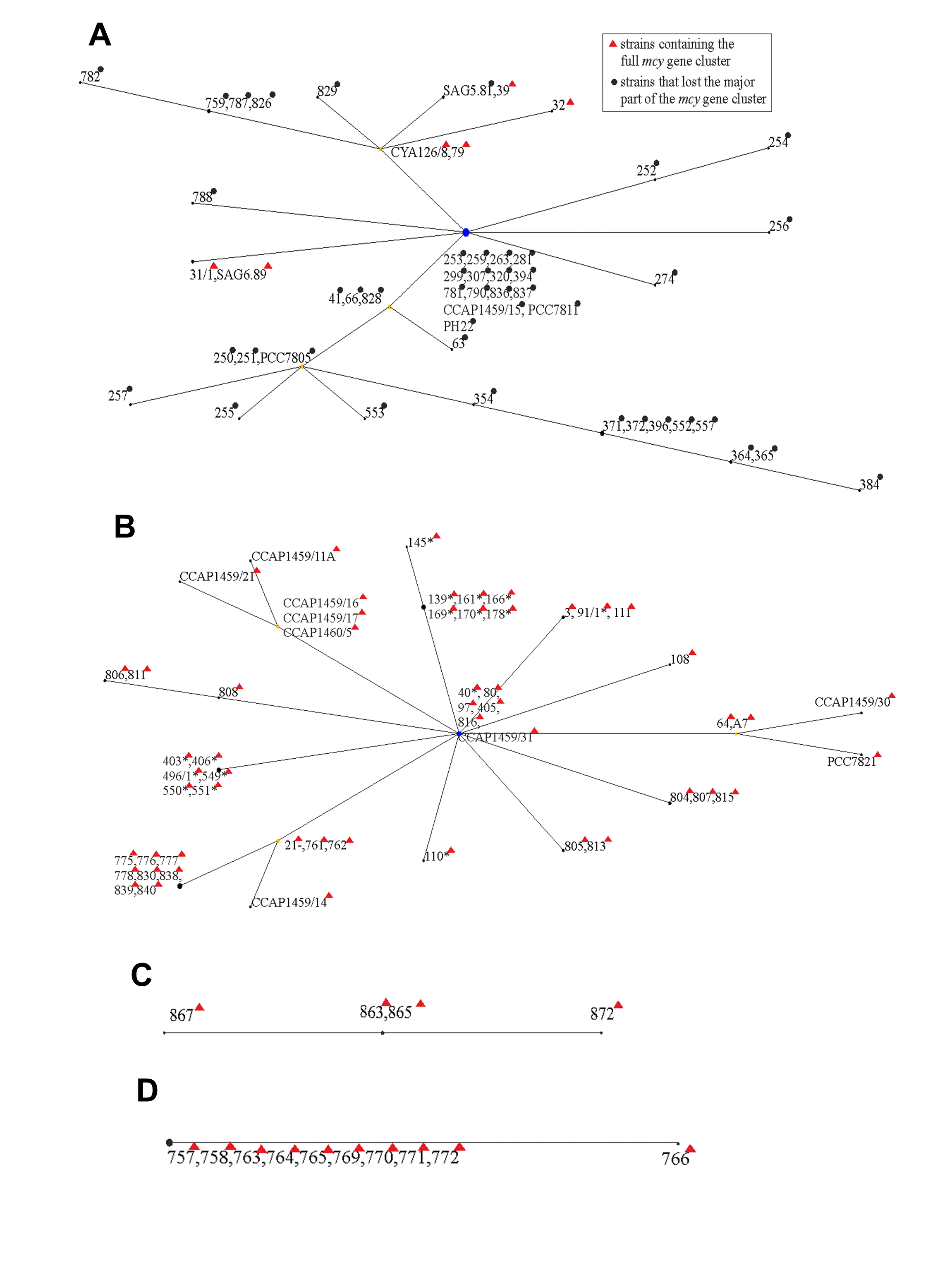
**

**Suppl. Figure 2.** MLST diagrams of clonal complexes of strains. Note that 20 singletons and 13 STs were not included but listed in the following: Lineage 1 (No2A; 760); Lineage 1A (No390; 277); Lineage 2 (No260, 779, 814); Lineage 2A (CCAP1459/36; No822; 873); Lineage 3 (No704; 707; 708; 710; 711; 712; 713); Lineage 4 (No82; 83/2; 67).

**A**, Group 1: No. Isolates = 51; No. STs = 23; Predicted founder = ST8 (Representing Lineage 1 (n = 42) and lineage 1A (n = 9) as shown in Figure 1B);

**B**, Group 2: No. Isolates = 53; No. STs = 20; Predicted founder = ST17, *, strains that have been found containing the whole mcy gene cluster but found inactive(Representing Lineage 2 (n =53) as shown in Figure 1B);

**C**, Group 3: No. Isolates = 4; No. STs = 3; Predicted Founder = ST55 (Representing Lineage 2A (n = 4) as shown in Figure 1B);

**D**, Group 4: No. Isolates = 10; No. STs = 2; Predicted Founder = None (Representing Lineage 2A (n =10) as shown in Figure 1B).

**Suppl. Figure 3.** Ratio of phycocyanin/phycoerythrin (PC/PE) of (A) green- and (B) red-pigmented lineages as identified in Figure 1B. For both pigmentation types subgroups are significantly different (Kruskal Wallis ANOVA on ranks; p<0.001). Superscripts indicate homogeneous subgroups as identified through post-hoc pairwise comparison (Dunn’s method; p < 0.05).

**Suppl. Figure 4.** Relative abundance of peptides of eight different peptide groups (I-VIII) among strains from different lineages as depicted in Table 4.


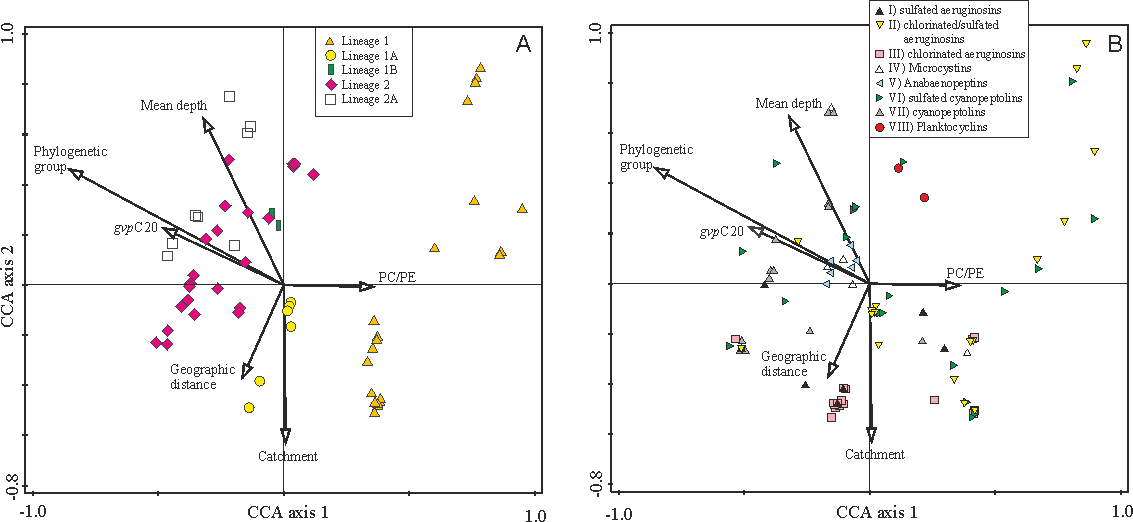


**Suppl. Figure 5.** CCA of peptide abundance (n = 95) recorded among 60 sequence types (recorded from 134 strains) in dependence on the environmental variables phylogenetic lineage, PC/PE ratio, gvpC^20^, mean depth, catchment area, geographical distance as identified by the manual forward selection procedure. **A.** strains vs variables plot; **B**. peptides vs variables plot. The peptides were numbered as shown in Suppl. Table 2. The influence of CCA axis 1 and 2 was highly significant (p < 0.002). In panel A, Orange triangle = lineage 1; Yellow circle = lineage 1A; Green rectangle= lineage 1B; Pink diamond = lineage 2; White rectangle = lineage 2A; in panel B, orange = sulfated aeruginosins (group I), .2 - .13 (MH-SO_3_); yellow = chlorinated/sulfated aeruginosins (group II), .18 - .44 (Cl^-^; MH-SO_3_); pink = chlorinated aeruginosins (group III), .47 - .70 (Cl^-^); circle = MCs (group IV), .71 - .75; square = Anabaenopeptins (group V): .76 - .81; green = sulfated cyanopeptolins (group VI), .82 - .122 (MH-SO_3_-H_2_O); underlined = cyanopeptolins (group VII), .126 - .150 (MH-H_2_O); Planktocyclins (group VIII), .158 - .159 (Planktocyclins).


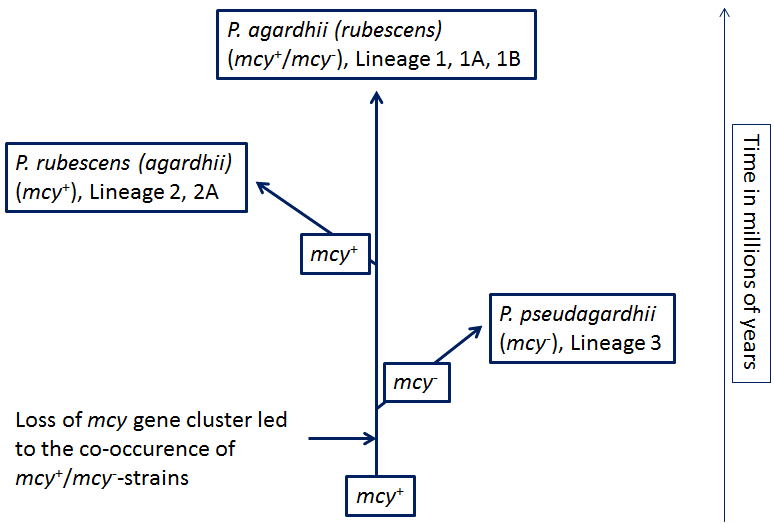


**Suppl. Figure 6.** Scheme on inheritance of the *mcy* gene cluster in *Planktothrix* spp. in accordance with the proposed lineages (Figure 1B).

***References:***
